# Advancing Posttraumatic Stress Disorder Diagnosis and the Treatment of Trauma in Humanitarian Emergencies via Mobile Health: Protocol for a Proof-of-Concept Nonrandomized Controlled Trial

**DOI:** 10.2196/38223

**Published:** 2022-06-15

**Authors:** Janaina V Pinto, Caroline Hunt, Brian O'Toole

**Affiliations:** 1 Faculty of Medicine and Health The University of Sydney Sydney Australia; 2 Sync Body-Brain Health Brisbane Australia; 3 School of Psychology Faculty of Science The University of Sydney Sydney Australia

**Keywords:** posttraumatic stress disorder, PTSD, trauma, humanitarian, emergencies, mobile health, mHealth, technology, neuroscience, electrophysiology, electroencephalogram, EEG, cognition, health system, biometric, health application, mental health, cognition, trauma, health intervention, mobile phone

## Abstract

**Background:**

Decentralized health systems in low- and middle-income countries (LMICs) affected by humanitarian crises lack resources and a qualified workforce to attend to the overwhelming demand for mental health care in emergencies. Innovative approaches that are safe, cost-effective, and scalable are needed to address the burden of traumatic stress caused by emergencies. High mobile phone ownership rates combined with the precision of neural, cognitive, and biometric measures of trauma and their feasible integration with artificial intelligence makes digital app interventions a promising pathway to promote precision diagnosis and high-impact care.

**Objective:**

This study aimed to advance methods for the objective diagnosis and treatment of trauma in emergencies across LMICs by examining neural, cognitive, and biometric markers and the efficacy of the eResilience app, a neuroscience-informed mobile health mental health app intervention, via changes in clinical symptomatology, cognitive performance, and brain activity.

**Methods:**

Trauma-exposed African refugees residing in Australia were selected for this study. A research software version of the eResilience app with advanced monitoring capabilities was designed for this trial. Participants completed the eResilience app at home during a 7-day period. Clinical, cognitive, and electrophysiological data were collected at baseline, along with posttest measurements to examine biomarkers of trauma and the efficacy of the proposed digital intervention for the treatment of trauma and its potential outcomes, including depression, anxiety, physical symptoms, self-harm, substance misuse, and cognitive impairment. In addition, biofeedback, well-being, and subjective stress data points were collected via the app during the treatment week, followed by clinical interviews at 1, 3, 6, and 12 months after the intervention.

**Results:**

Data collection was conducted between 2018 and 2020. A total of 100 participants exposed to war were screened; 75 (75%) were enrolled and assigned to a trauma-exposed control (38/75, 51%) or posttraumatic stress disorder condition (37/75, 49%); and 70 (70%) completed all baseline, treatment, and posttest assessments. A total of 89% (62/70) of those who completed the intervention opted to enroll in the 3-, 6-, and 12-month follow-ups. Data collection is complete. As of May 2022, the results of all proposed analyses are being prepared for publication. If proven efficacious, this proof-of-concept clinical trial will inform fully powered randomized clinical trials in LMICs to further develop artificial intelligence–powered, app-based diagnostic and prognostic features and determine the app’s cross-cultural efficacy for the treatment of trauma in emergency settings.

**Conclusions:**

This protocol provides researchers with a comprehensive background of the study rationale, a detailed guideline for replication studies interested in examining the feasibility and efficacy of the eResilience app across varied demographics, and a robust framework for investigating low-cost objective diagnostic markers in mental health interventions. Methodological limitations and suggestions are also provided.

**Trial Registration:**

Australian New Zealand Clinical Trials Registry ACTRN12616001205426; https://tinyurl.com/yckwc4d7

**International Registered Report Identifier (IRRID):**

RR1-10.2196/38223

## Introduction

### Background

Nations worldwide face the continuous challenge of addressing the complex and varied consequences of past, current, and evolving humanitarian emergencies. The United Nations Office for the Coordination of Humanitarian Affairs estimates that there are currently 234 million vulnerable people across 56 countries affected by war and conflict, climate change, hunger, and the COVID-19 pandemic [1]. Humanitarian emergencies, whether man-made, natural hazards, or public health crises, are often characterized by loss of life, mass-scale displacement, security risks for aid workers, economic crises, political tension, and wearying of public health systems leading to constraints in the provision of large-scale health care for the affected population. Although the psychological aftermath of disasters is still overlooked in emergency responses, the growing global recognition of the burden of mental health [2] disease has prompted many scientific studies on the long-term implications of psychological distress in humanitarian crises. This burden is well-documented across the domains of justice, peace and reconciliation [3-7], policy and legislation [8], and its impact on the economy [9,10], with a presumably accentuated economic impact in low- and middle-income countries with fragile health systems [11]. Therefore, understanding and addressing the mental health outcomes of disasters can not only promote the health of populations affected by adversity but also alleviate the burden across all societal domains affected. However, to address such issues, innovations are needed to resolve some of the core pressing clinical challenges faced by humanitarian actors, such as discrepancies in subjective diagnostic tools and costly mental health treatment protocols that require specialized clinicians and that, for the most part, lack cultural sensitivity.

### Mental Health Apps for Humanitarian Emergencies

Accelerated by the COVID-19 pandemic, investments in digital health are increasing markedly. In 2020 alone, >90,000 new health apps emerged in a market that already surpasses 350,000 apps, of which 22% account for mental health apps promoting the management of behavioral disorders via cognitive behavior therapy (CBT) strategies, mindfulness, meditation, stress and anxiety management, and sleep hygiene practices and monitoring [12]. However, despite the fast growth in the digital health field, very few apps are suitable or have been designed for use in emergency response as promising innovations are, for the most part, incentivized by the monetization of sophisticated trends in digital technologies such as artificial intelligence, automated therapies, and virtual reality–based treatments [13].

Although not trauma-focused, an example of a successful digital adaptation of CBT-based care for humanitarian settings is Problem Management Plus (PM+), an initiative by the World Health Organization as part of the mental health Gap Action Programme [14] developing mental health solutions for low- and middle-income countries that can be scaled to attend to large populations. PM+ was designed to promote brief psychological interventions for adults with sessions once per week during a 5-week period, promoting behavioral, stress management, and everyday problem-solving tools [15]. Emerging data from feasibility trials and randomized controlled trials in Pakistan [16-18], Nepal [19,20], and Kenya [21,22] indicate that PM+ is promising for the effective management of psychological distress, including trauma symptoms, and problems in daily life via app in emergency settings. Alongside additional studies on PM+ taking place in Europe to attend to the needs of refugees and asylum seekers [23-27], the European Union STRENGTHS program is also a promising initiative examining the efficacy of PM+ in attending to the psychological needs of Syrian refugees across Europe and Middle Eastern countries [28]. However, developers emphasize that PM+ is a low-intensity intervention for adults with depression, anxiety, or stress in areas affected by adversity and is not designed to address the full range of challenges brought on by adversity; thus, it is recommended for use in conjunction with other support systems. Moreover, the World Health Organization stresses that PM+ is not suitable to address suicidality and other severe mental, neurological, or substance use disorders. Further limitations reported include challenges in scalability in Kenya [29] and cost-efficacy in Pakistan [30].

Overall, only 2 studies on app-based interventions designed specifically for trauma in a humanitarian context were identified. A randomized clinical trial examined the efficacy of Sanadak, a CBT-based self-help trauma app in Arabic for Syrian refugees [31]. In comparison with a control condition, the Sanadak app was not more effective in reducing posttraumatic stress disorder (PTSD) symptomatology and, furthermore, it was not likely to be a cost-effective solution. An additional study protocol has been recently published aimed at conducting an examination of another CBT-based trauma app for Syrian refugees in Germany, but the results have not yet been published [32].

### Study Objectives and Relevance

This study was focused on 2 primary objectives. First, it aimed to investigate novel neural and cognitive diagnostic and prognostic markers of clinical and subclinical PTSD with the potential for future integration in portable technology. We proposed that objective markers, when integrated into artificial intelligence, could aid lay humanitarian actors in the fast and accurate screening of individuals in need of care, guide best practices, assist with precision impact evaluation, and serve as predictive measures in prevention initiatives. Second, this proof-of-concept study introduced a trauma-focused digital mental health intervention, the eResilience app. Although the intervention’s clinical rationale presents a transdiagnostic approach targeting basic physiology and neurocircuitry affected by stress, hence potentially having clinical utility to address a range of mental health conditions, this study examined its feasibility as a primary treatment for clinical and subclinical PTSD. The examination outcome assessment process for the intervention was based on psychological, cognitive, and neurobiological systems via (1) electrophysiological activity during a state of rest, (2) neuropsychological testing, and (3) clinical symptomatology.

### eResilience App

#### Core Features

The eResilience app was proposed as a stand-alone intervention. The clinical curriculum is personalized for communities living under the poverty line, categorized by earning <US $5 per day. Moreover, it is low-intensity, without the involvement of specialists; it is trauma-focused, based on core components of complex trauma interventions including safety, self-regulation, self-reflective information processing, relational engagement, and positive affect enhancement [33]; it adheres to nonexposure practices as a safety measure; it is standardized in a toolkit format; it is 10 hours long and designed for completion during a short period of 5 to 7 consecutive days; it uses a clinical multimodal approach combining a diversity of clinical tools; it is culturally adaptable and neuroscience-informed; and, finally, it is designed to scale and reach large vulnerable populations.

#### Clinical Rationale

In alignment with the core components of complex trauma interventions, the curriculum was based on three clinical aims established for the eResilience protocol: (1) the creation of a safe and personal therapeutic space to hone skills of autonomic nervous system (ANS) control and awareness, (2) the enhancement of core cognitive functions affected by trauma, and (3) building or restoring relational engagement. The clinical tools in the curriculum that aim to build ANS awareness and control are rooted in theoretical and biological processes in the sympathetic and parasympathetic branches of the nervous and somatosensory systems. The method includes (1) building body awareness skills, (2) training one’s ability to shift awareness between somatic sensations, and (3) identifying internal and external resources for top-down and bottom-up self-regulation, altogether aiming to promote a sense of safety that has been disrupted by trauma in the body and the environment. The clinical tools selected for building ANS awareness and control included breath work, progressive muscle relaxation, biofeedback, rhythm-focused exercises (music therapy, binaural beats, and bilateral tapping), yoga nidra, guided imagery, and grounding techniques. Therapeutic tasks primarily targeting cognitive function in the prefrontal cortex introduce the practice of exteroception skills, including engaging attention shifting from external surroundings, such as objects and sounds, and a short written task (which may also be adapted to accommodate the needs of individuals with low literacy levels) to identify internal and external resources for coping with adversity, as well as self-reflective information processing, positive affect, meaning making, and gratitude practice aimed at enhancing overall executive function capacities disrupted by trauma. For relational systems, selected tasks were informed by the biological principles and neural circuitry of social behavior and included the introduction of subjective and real-life safe interpersonal experiences, support system identification, building a sense of purpose in the community, and engagement in altruistic behavior, altogether aiming to promote environmental safety, interpersonal relatedness, planning, decision-making, initiation, and pleasure in the context of social interactions.

#### Software

In total, 3 software versions of the eResilience app have been created. The first prototype, also known as a minimum viable product, along with its first field version designed for groups, was created for and piloted by an international charity in West Africa. For this clinical trial, the eResilience research version 1.0 (Multimedia Appendix 1) was created and optimized to include clinical trial safety measures, accommodate individual participant use, and collect biometric and self-report data during the intervention week. The software specifications are detailed in the *Methods* section.

### Research Questions and Hypotheses

On the basis of the evidence presented for anomalies in electrophysiological and cognitive performance in PTSD, this study questioned whether resting-state electrophysiological activity and neuropsychological performance were reliable objective measures for diagnosing clinical and subclinical PTSD and predicting intervention outcomes. It was hypothesized that, at baseline, (1) the PTSD cohort would present statistically significant differences in quantitative electroencephalograms (EEGs) compared with the controls and that (2) the PTSD group would perform more poorly than the control participants on the cognitive tests. Moreover, in questioning the feasibility of the proposed treatment for clinical and subclinical PTSD, we hypothesized that, at posttest measurements, the intervention would (1) reduce PTSD severity according to the Clinician-Administered PTSD Scale for the Diagnostic and Statistical Manual of Mental Disorders, Fifth Edition (DSM-5; CAPS-5), Past-Month Edition, between baseline and posttest measurements (3-, 6-, and 12-month follow-up time points) and (2) improve participant performance in cognitive measures.

## Methods

### Ethics Approval

The study was approved by the Human Research Ethics Committees of the University of Sydney and the University of the Sunshine Coast, Australia. This trial was conducted in compliance with the ethics committee approval conditions, the National Health and Medical Research Council Statement on Ethical Conduct in Human Research (2007), and the Note for Guidance on Good Clinical Practice (CPMP/ICH-135/95).

### Study Design

**Figure 1:**
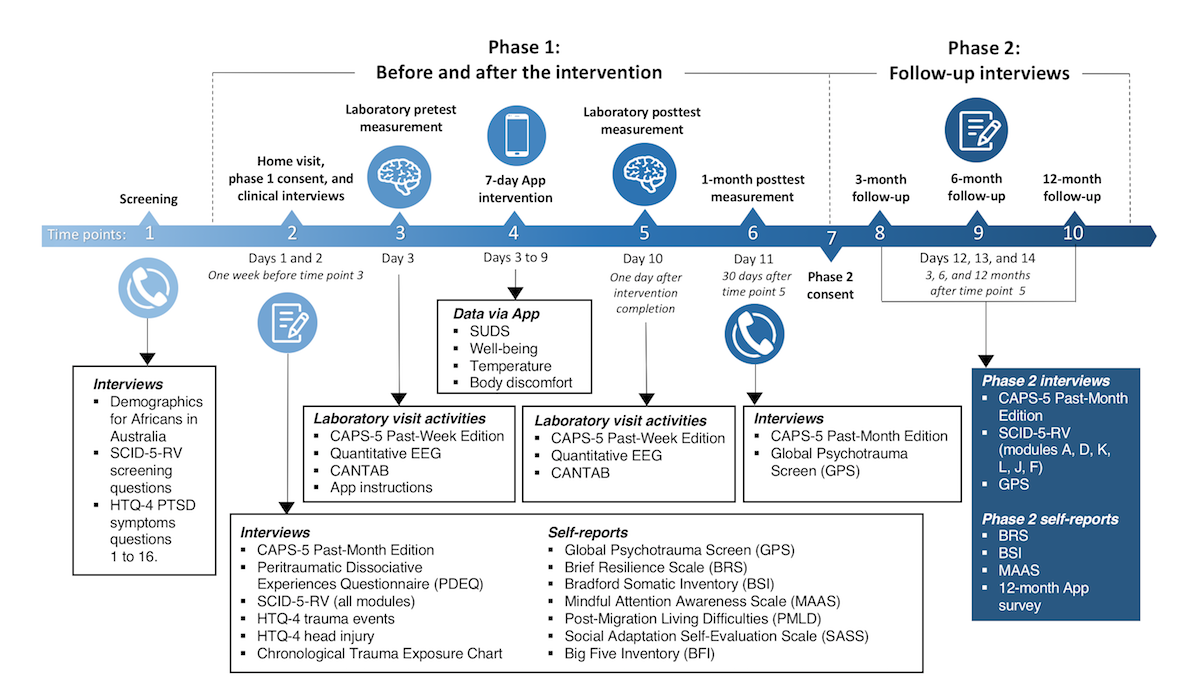
Study design. CANTAB: Cambridge Neuropsychological Test Automated Battery; CAPS-5: Clinician-Administered Posttraumatic Stress Disorder Scale for the Diagnostic and Statistical Manual of Mental Disorders, Fifth Edition; EEG: electroencephalogram; HTQ-4: Harvard Trauma Questionnaire-4; PTSD: posttraumatic stress disorder; SCID-5-RV: Structured Clinical Interview for the Diagnostic and Statistical Manual of Mental Disorders, Fifth Edition, Research Version; SUDS: Subjective Units of Distress Scale; TBI: traumatic brain injury.

This proof-of-concept study was designed as a nonrandomized controlled trial, including 10 time points across the 2 study phases. The first phase involved a 10- to 11-day commitment, comprising intake (time point 1), 1 or 2 baseline home visits to complete clinical interviews (time point 2, within 2 weeks of time point 1), a baseline university laboratory visit (time point 3, after 7 days of time point 2), the eResilience app intervention (time point 4, for 7 consecutive days starting the day of time point 3 upon laboratory data collection completion), a posttest university laboratory visit (time point 5, the day after time point 4 completion), and the 1-month follow-up (time point 6, after 30 days of the time point 5 laboratory visit). The second phase of the study included a new informed consent (time point 7, within 2 months of completing time point 6) to collect 3-, 6-, and 12-month follow-ups (time points 8, 9, and 10). The interventions at each time point are summarized in Figure 1. In total, participants in both phases of the study committed between 13 and 14 days to complete the trial within a 12-month period.

### Electrophysiology Paradigm and Acquisition

#### Paradigm

Quantitative EEG signals were collected in a sound-attenuated room during a resting state with 4-minute eyes-open and 4-minute eyes-closed paradigms and a 1- to 5-minute break between recordings to check and fix impedance as needed. The participants were instructed to fixate on a dot in the center of a television monitor during the eyes-open collection and rest quietly during the eyes-closed paradigm.

#### Participant Protocol

The participants followed instructions to not consume alcohol, caffeinated drinks, or nicotine on the day of the quantitative EEG recordings. Moreover, they were instructed to wash their hair with only shampoo the evening before the laboratory visits and avoid conditioners, gels, oils, and hair spray to increase electrode adherence to the scalp.

#### Data Acquisition and Export

Data were acquired at a sampling interval of 4000 µS and sampling rate of 250 Hz using the Electrical Geodesics’ (EGI) Geodesic EEG System 400 [34]. A scalp EEG was recorded from 32 electrode sites according to the standard 10-20 International System [35,36] with the 32-channel HydroCel Geodesic Sensor Net which includes 2 link mastoids and 4 electrodes for vertical and horizontal eye movement tracking. Impedance was kept <5 kΩ. Raw EGI data were exported in a binary format with integer precision for preprocessing in the BrainVision Analyzer Software (version 2.2.1) [37]. A software solution was installed to read the original Cartesian electrode positions used in the EGI system.

### Clinical Interviews

Several diagnostic clinical interviews as well as psychosocial and self-report questionnaires were selected for this study. All clinical interviews were conducted by a masters-level clinician with 12 years of experience conducting trauma assessments with African refugee communities (JVP) and a trained clinical research assistant with experience collecting and evaluating trauma assessments of 400 refugees across Uganda (Elsa Goninon). Challenging diagnostic decisions were discussed among senior researchers in the team (CH and BOT).

The CAPS-5 was chosen as a primary outcome measure for PTSD, possessing excellent convergent and discriminant validity (κ=0.84) [38], interrater reliability (κ=0.78-1.00), and test-retest reliability (κ=0.83). The full version of the Structured Clinical Interview for the DSM-5, Research Version [39], was collected face to face for diagnostic assessments of additional psychiatric conditions, including mood, anxiety, obsessive-compulsive disorder and related conditions, substance use, sleep, feeding and eating, somatic symptoms and related conditions, and externalizing disorders. To the best of our knowledge, there are currently no studies examining the psychometric properties of the Structured Clinical Interview for the DSM-5, Research Version; however, the clinical version of the Structured Clinical Interview for the Diagnostic and Statistical Manual of Mental Disorders has demonstrated excellent interrater reliability for most diagnoses (κ values reported to be ≥0.75, diagnostic sensitivity >0.70, and specificity >0.80) [40]. The Global Psychotrauma Screen (GPS) [41] is a new, 22-question screening instrument designed to identify reactions to a potentially traumatic event or severe stressor within the month before assessment, demonstrating robust concurrent validity and good internal consistency, with acceptable Cronbach *α* coefficients across several studies [41-43]. The GPS data collected in this study will be used to examine the clinical validity of the instrument among refugees.

Additional clinical interviews included items 1 to 6 of the Peritraumatic Dissociative Experience Questionnaire [44], a self-report questionnaire that assesses dissociation (test-retest reliability: *r*=0.77; internal consistency: *α*=.89) [45]; the Harvard Trauma Questionnaire (HTQ) [46] to assess torture, trauma, traumatic brain injury, and trauma-related symptoms (test-retest reliability: 0.89; interrater levels: 0.098; test-retest reliability: 0.92; internal reliability for varied ethnic backgrounds, eg, 0.98 for Indochinese groups) [46]; the Social Adaptation Self-Evaluation Scale [47], a 20-item questionnaire measuring social motivation and behavior (Cronbach *α*=.74; no significant changes in test-retest reliability between the 2 first releases of the measure; *P*>.05) [47]; the Post-Migration Living Difficulties [48,49], a 17-item checklist assessing postmigration difficulties encountered by refugees proposed to serve as a predictor for poor mental health in refugee groups [50-52]; the Mindful Attention Awareness Scale [53], a 15-item self-report survey designed to assess levels of dispositional trait mindfulness (internal consistency among African American populations: *α*=.90 [53]; convergent validity negatively correlating with a measure of overall psychological distress [*r*=−0.38] and positively correlating with a measure of psychological flexibility [*r*=0.45] [54]); the Big Five Inventory [55], a 44-item self-report scale used to assess the prevalence of the 5 dimensions of personality (internal consistency among African American populations across the five subscales: agreeableness, *α*=.70; extroversion, *α*=.83; conscientiousness, *α*=.79; openness, *α*=.72; and neuroticism, *α*=.70) [56]; the Brief Resilience Scale [57], a 6-item questionnaire designed to assess one’s ability to bounce back from stress (internal consistency: *α*=.80-.91; 1-month test-retest reliability interclass correlation coefficient=0.69; convergent and predictive validity positively correlating with measures of active coping, positive reframing, and planning [*r*=0.27-0.42] and negatively correlating with a range of poor health-related outcomes such as perceived stress, negative affect, anxiety, and depression [*r*=0.34-0.60]) [57]; and, finally, the Bradford Somatic Inventory [58], a multiethnic inventory that assesses the presence and severity of somatic symptoms related to anxiety and depression (internal consistency: *α*=.86 [59] and good test-retest reliability within a British care population).

Finally, 3 instruments were created for this study. The Demographics for Africans in Australia (Multimedia Appendix 2) is a 27-item questionnaire designed to collect demographic data specific to the experiences of African refugees resettled in Australia. The Chronological Trauma Exposure Chart (Multimedia Appendix 3) was created to supplement the assessment of trauma history. Considering that many participants in the communities selected for recruitment are commonly not aware of their exact birth date and that the long-term exposure to traumatic experiences during extended years of war presents a challenge in remembering precise years and dates, the chart contains a table with a range of years of interest on the x- and y-axes, x representing the participant’s year of birth and y outlined by year in accordance with the main war-related events in each country. This way, the chart allows researchers to accurately identify the year of a described traumatic exposure that may have only been recalled according to a particular event in the war (eg, the fragile peace period in Liberia in 1997 or the state conflicts in 2012 in Sudan), thus pragmatically allowing the researchers to immediately identify and confirm with the participant their estimated age at the time of the reported event. The second half of the chronological chart attempts to aid in identifying the age, frequency, and duration of 8 traumatic experiences common among the target participants, including exile, bush hiding, malnourishment, sexual assault or rape, physical torture, cannibalism, and fighting in the war as adults or children. Finally, to examine the long-term qualities of the intervention according to both quantitative and qualitative subjective reports, a 7-item app survey (Multimedia Appendix 4) was developed for administration at the 12-month follow-up for participants who completed all stages of the study. The survey asks participants if they practiced, told others, or taught others the skills they learned during the previous year, including details such as frequency and mode of practice, recall of favorite activities, and perceived life improvement. In total, 2 exteroceptive questions also ask participants to check what resources they have often found most helpful throughout life to overcome adversity and the role religion may have played in adapting to difficult life situations.

### Cognitive Tests

The Cambridge Neuropsychological Test Automated Battery [60] is a computerized and standardized assessment administered via tablet to measure the key cognitive processes of (1) information input into the brain, such as attention and processing speed; (2) information representation in the brain, such as memory; and (3) use of stored information to guide behavior, also known as executive function. The Cambridge Neuropsychological Test Automated Battery system has been used in other trials examining cognitive function in PTSD [61-69]. The tests are language-independent and suitable for cross-cultural experiments. The following tests were selected for this study for identification of diagnostic and prognostic cognitive markers: Motor Screening Task, Reaction Time, Rapid Visual Information Processing, Paired Associates Learning, Delayed Match to Sample, Spatial Working Memory, One Touch Stockings of Cambridge, and Emotion Recognition Test.

### Intervention

At time point 3, the participants were individually taken through a 30-minute Microsoft PowerPoint introduction to the eResilience app. The session included handling a take-home kit containing printed safety guidelines, a smartphone with software, soundproof headphones, biofeedback equipment for temperature measurements, and the printed app response booklet (Multimedia Appendix 5). The participants were guided step by step on how to set up the biofeedback equipment at home and open and use the app and its features and provided with safety briefings and a short demonstration of each clinical task. Finally, the researchers programmed the software in the presence of each participant to activate the daily app alarm at the preferred time as chosen by the participant in the following 7 days. Once programmed, the smartphone app activated daily reminder alarms at 1 hour and 15 minutes before the time specified to commence the program each day. The participants were instructed to leave the phone always plugged at home and with the volume on. For time point 4, all participants took the app kit home and followed instructions on the smartphone screen to complete a sequence of daily tasks for 7 consecutive days. During the treatment week, the participants completed the tasks by themselves and at their own pace with a time frame ranging between 90 and 100 minutes, given the pause button option across all intervention blocks. Access to the tasks for each day was unlocked every 24 hours at the hour selected by the participant during time point 3 to prevent participants from potentially attempting to complete more than one session per day in the same 24-hour period.

### Software Specifications

The eResilience app research version (Multimedia Appendix 1) designed for this study includes 2 user dashboards: one for researchers and the second one for study participants. The researcher dashboard presents features to (1) add participants and schedule their intervention commencement time; (2) a user-friendly database of participant activity; (3) a management system for the allocation of smartphones and other research equipment; and (4) a real-time participation monitoring feed including mobile SMS text message notifications each time a participant commences, concludes, or skips a task. Once the researcher launched the participant dashboard on the app, a secure log-in was needed to switch back to the researcher dashboard, thus making it impossible for participants to make changes. The participant dashboard home screen displayed a user-friendly layout with a simple 1-click motion to start each of the 7 days of the intervention. Although it displayed all 7 buttons for the 7 days of the intervention, only the current day was activated for access each day. This feature unlocked the curriculum every 24 hours at the daily time stipulated by the participant at baseline, preventing participants from attempting to complete the curriculum in <7 days. A daily alarm set by the researcher reminded the participant to complete the tasks at 1 hour and at 15 minutes before the elected starting time.

Once the intervention was launched, the clinical tasks were presented via visual and audio instructions recorded in a pitch-controlled, text-to-speech software. Advanced features to promote participant safety included well-being, body discomfort, and stress checks added at the start and end of the day and in between blocks of exercises, automating SMS text messages that were sent to researchers on standby whenever a participant reported elevated levels of distress during the intervention. As part of the study design, the software also presented integration for external biofeedback equipment for temperature check-ins as part of the objective stress measurements. The data set collected via software during the intervention week included time-tracking features to register precise individual participation (eg, when a clinical task had been paused, skipped, or not played). Data were secured and accessed via a password-protected researcher account.

### Recruitment

Participants were recruited in collaboration with the following local African community organizations in the state of Queensland: the *Federation of Liberian Communities in Australia*, the *Liberian Association of Queensland*, the *Congolese United for Peace and Reconciliation in Australia*, *Together We Are Powerful Inc*, and the *South Sudanese Youth Council*. Further recruitment support was also received from three nonprofit charities: *Second Chance Africa, Sync Body-Brain Health,* and *Welcoming Australia*. Building rapport with community leaders was imperative for successful recruitment, managing cross-cultural expectations, and ensuring that the project aim was well understood before approaching community members. Each community leader held meetings with researchers and other nonprofit staff for several months to discuss the details of the project. They also visited the neuroscience laboratory to understand all the data collection procedures before introducing the project to their community members. All African community leaders mutually agreed to assist the study motivated by the project’s potential outcome to disseminate effective trauma care worldwide across regions of disaster via our supporting charities and sponsored 7 recruitment social events to introduce the opportunity to their members. The events included free cultural activities such as live music, sports, dance, and traditional food proposed by the community leaders. During the events, the researchers, along with the community leaders, adhered to the approved study advertisement materials, distributed handouts of the participant information statement (Multimedia Appendix 6) to provide information about the study, and provided opportunities for those interested to ask questions regarding participation. An expression-of-interest sign-up sheet was also used during events for contacting prospective participants. Most participants who completed the project also referred other friends and family members to participate in the study.

### Study Phase 1 Eligibility and Consent

#### Inclusion Criteria

Participants were eligible if they were Liberian, Congolese, or Sudanese refugees who had migrated to Australia after the age of 18 years. All participants were required to have English proficiency and have fled emergency in Africa between 1989 and 2018. The participants were divided into two groups: a PTSD group, including clinical and subclinical PTSD cases according to the diagnostic specifications outlined in Textbox 1, and a trauma-exposed control group who did not meet the criterion for clinical or subclinical PTSD or other mental health disorders.

**Posttraumatic stress disorder (PTSD) diagnostic inclusion criteria.**

**Inclusion criteria**

- Diagnostic and Statistical Manual of Mental Disorders, Fifth Edition (DSM-5) threshold: Clinician-Administered PTSD Scale for the DSM-5 (CAPS-5) criteria A+B+C+D+E+F+G
- DSM-5 subthreshold: CAPS-5 criteria A+(at least one threshold symptom at B, C, D, and E)+F+G
- DSM-5 subsyndromal: CAPS-5 criteria A+B+(meets criteria for cluster C or D or E or any 2 of the 3 clusters, but not C+D+E combined)+F+G
- DSM-5 other stress or trauma-related disorders: CAPS-5 criteria A+F+G (and no overlap with DSM-5 Subsyndromal cases)
- Diagnostic and Statistical Manual of Mental Disorders, Fourth Edition (DSM-IV) threshold: Clinician-Administered PTSD Scale for the DSM-IV (CAPS-4) criteria A+B+C+D+E+F
- DSM-IV subthreshold: CAPS-IV criteria A+(at least one threshold symptom at B, C, and D)+E+F
- DSM-IV subsyndromal: CAPS-4 criteria A+B+(C or D)+E+F
- DSM-IV Harvard Trauma Questionnaire (HTQ) risk screen: HTQ mean score of items 1 to 16 ≥2.5 (symptomatic for PTSD)
- DSM-IV threshold: CAPS-IV criteria A+B+C+D+E+F
- DSM-IV subthreshold: CAPS-4 criteria A+(at least one threshold symptom at B, C, and D)+E+F

#### PTSD Group Diagnostic Inclusion Criteria

Considering the reported limitations of the Diagnostic and Statistical Manual of Mental Disorders in identifying trauma among refugee populations [70], we broadened the PTSD symptom profile in the inclusion criteria (Textbox 1) to include clinical (threshold) and subclinical (subthreshold, subsyndromal, other stress or trauma-related disorders, and risk screening) profiles. Researchers ensured that two core characteristics were met for all participants enrolled in the PTSD group: (1) the presence of trauma symptomatology in response to a humanitarian event and (2) the reported trauma-related cause significant life impairment.

#### Exclusion Criteria

Exclusion criteria for both groups included a self-reported severe medical condition, genetic disorder, or concurrent drug or alcohol abuse or dependency within the month before the intervention trial.

Additional exclusion criteria for the PTSD group included current use of psychotropic medication or use within the 2 months preceding the intervention, concurrent psychotherapy for PTSD, and acute risk of suicide or homicide.

Considering the various levels of trauma symptomatology proposed for the PTSD group, additional exclusion criteria for the trauma-exposed control group included not meeting the threshold, subthreshold, subsyndromal, or other stress- or trauma-related disorders criteria on either the Diagnostic and Statistical Manual of Mental Disorders, Fourth Edition (DSM-IV), or the DSM-5 as well as not meeting PTSD risk or symptom criteria on the HTQ.

#### Study Phase 1 Consent

All participants enrolled provided oral and written consent and signed a participant consent form at time point 2 (Multimedia Appendix 7).

### Study Phase 2 Eligibility and Consent

Participants were contacted 30 days after completion of the 1-month posttest measurement, invited to participate in the study phase 2—involving 3-, 6-, and 12-month follow-up interviews—and given a copy in person or via email of the second participant information statement (Multimedia Appendix 8). Eligibility to participate in the second phase of the study included having completed the first phase. Those who opted to join provided oral and written consent and signed the second participant consent form (Multimedia Appendix 9) at time point 7.

### Statistical Analyses

#### Overview

Study measures will be compared between the PTSD and control group participants. Descriptive statistics and 2-tailed *t* tests will be used to describe the sample and compare changes between groups.

All time-domain quantitative EEG recordings will be plotted into a frequency power spectrum using fast Fourier transform. The grand average for each segment of data for the control and experimental conditions will be calculated at pre- and posttest measurements across both paradigms to generate topographical distributions of spectral power values in the delta, theta, alpha, peak alpha, beta, peak beta, and gamma frequency bands. The results will be presented in the form of power values (uV2).

On the basis of the respective data distribution, McNemar nonparametric tests, *t* tests, and repeated ANOVAs will be used for all quantitative EEG, neuropsychological, and clinical data collected across time points to identify statistically significant changes after the intervention. Changes in selected variables of both groups between the baseline and postintervention conditions will be investigated via additional independent sample *t* tests and Wilcoxon nonparametric tests. Linear regression analyses and chi-square tests will be used to identify predictors of treatment response between the subjective and objective variables. Further correlation analyses will be conducted to assess the relationship between clinical and cognitive variables in the experimental condition to explore associations between the app, PTSD symptomatology, and cognitive and electrophysiological variables. Additional post hoc analyses will be conducted as needed.

#### Statistical Power and Sample Size Estimation

The proposed sample size of 60 for this study (PTSD: 30/60, 50%; controls: 30/60, 50%) was based on the effect size computation of a quantitative EEG measure previously used to investigate the dimensional complexity of the EEG between the PTSD and control groups [71]. This produced a mean of 11.991 (SD 0.1760) for the non-PTSD group and of 11.1689 (SD 0.2437) for the PTSD group based on EEG leads that yielded significant results (frontal electrodes on both hemispheres Fp1, F8; central electrodeC4 and parietal electrode P4 on the right hemisphere; temporal electrodes T3, T4, T5, and T6 on the right and left hemispheres, and occipital electrode O1 on the left hemisphere) [49]. An SD estimate of 0.612 for the control and PTSD groups and a mean difference of 0.822 between the PTSD and control groups indicated an effect size of 1.343 and a 100% statistical power estimation with a sample size of 30 per condition. Clinical studies on PTSD have often achieved large effect sizes with samples of 30 patients per condition in accordance with the recommendations of the International Society of Traumatic Stress Studies Treatment Guidelines [72].

## Results

### Recruitment Results

**Figure 2:**
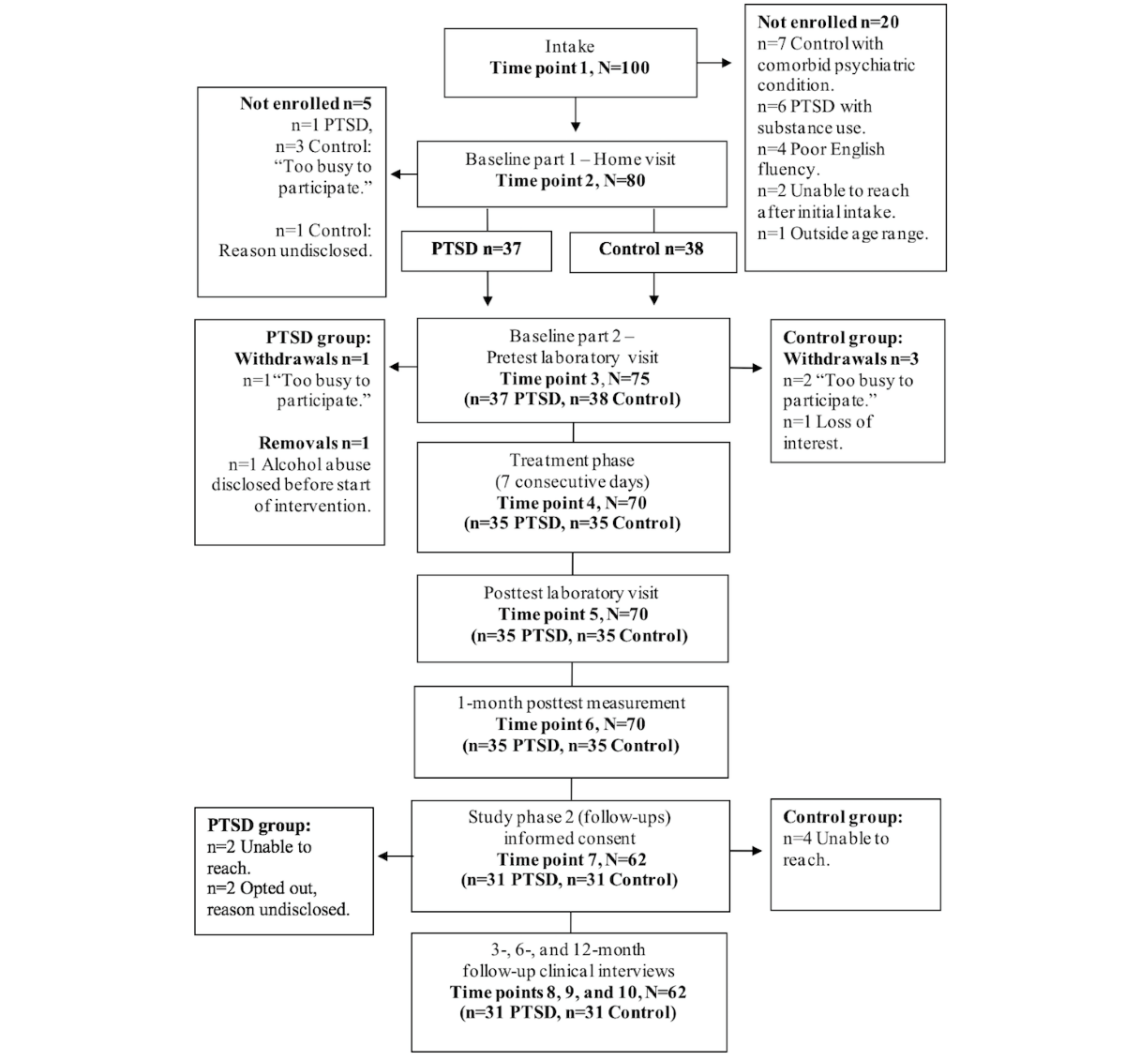
Study recruitment results flowchart.

For the recruitment results (Figure 2), at the first time point (time point 1), a total of 100 individuals were screened for this study, of whom 20 (20%) did not meet the inclusion criteria because they were control participants with comorbid psychiatric conditions (7/20, 35%); PTSD group participants with reported substance use (6/20, 30%); or participants between groups with poor English fluency (4/20, 20%), outside the target age range (1/20, 5%), or unable to be contacted (2/20, 10%).

In the first phase of baseline assessments, time point 2, a total of 80 participants consented and were fully assessed, and 75 (94%) chose to enroll in the intervention. All enrolled participants (75/75, 100%) completed the second baseline phase (time point 3) and took the eResilience app home to complete the intervention during the 7-day period (time point 4). In total, 7% (5/75) of the participants withdrew consent before commencing the intervention because of lack of time (4/5, 80%) and a disclosure of alcohol abuse (1/5, 20%); thus, their data will be removed from the analyses. All 70 participants who completed the intervention returned to the laboratory for postintervention assessments (time point 5) and completed the 1-month follow-up assessment (time point 6) via telephone. Upon completion of the 1-month follow-up, the participants were contacted and extended an invitation to participate in the 3-, 6-, and 12-month follow-ups for the study. In total, 89% (62/70) of the participants who completed the trial consented and opted to enroll again (time point 7). Of the 8 participants who did not join the follow-ups, 2 (25%) were part of the PTSD group and opted out without disclosing reasons, and 6 (75%) were unable to be reached because of changes in phone numbers, addresses, or email (PTSD: 2/6, 33%; controls: 4/6, 67%). All participants enrolled in the follow-ups (62/62, 100%) completed the interviews at the 3 time points (time point 8, time point 9, and time point 10). In summary, the final data sample includes 70 participants at baseline, 1-week, and 1-month posttest analyses and 62 participants in the 3-, 6-, and 12-month follow-ups.

### Participant Profiles

The final sample that completed the treatment phase (n=70) included 44% (31/70) men and 56% (39/70) women aged 18 to 54 years (mean 33.64, SD 10.54 years). The participants were Congolese (Democratic Republic of the Congo; 23/70, 33%), Liberian (22/70, 31%), and Sudanese (25/70, 36%) and altogether represented 26 African ethnic groups. The PTSD cohort (35/70, 50%) included 69% (24/35) of participants who met the full DSM-5 PTSD diagnostic criteria based on the CAPS-5, 14% (5/35) who met the DSM-5 subthreshold criteria, and 17% (6/35) who met the DSM-5 subsyndromal criteria, as detailed in Textbox 1. When translating the reported symptomatology of the PTSD cohort to the DSM-IV–based criterion, 63% (22/35) met the threshold, 34% (12/35) met the subthreshold, and 3% (1/35) met the subsyndromal criteria. On the basis of the DSM-IV HTQ risk screening, only 43% (15/35) of the PTSD group participants had scores indicating being symptomatic for PTSD. In the trauma-exposed control group (35/70, 50%), 40% (14/35) presented no PTSD symptoms, 23% (8/35) presented 1 mild symptom, and 9% (3/35) presented more than one mild symptom but no threshold symptoms. Moreover, 29% (10/35) of the controls presented one or more threshold symptoms but disclosed not being bothered by them. None of the control participants met the clinical or subclinical PTSD criterion. Most importantly, none of the control participants who presented trauma symptoms (21/35, 60%) reported levels of impairment or trauma-related distress.

### Data Collection Adaptations

The participants were asked to complete the GPS on their own at the end of the home visit. Nevertheless, upon noticing that several participants mistakenly responded to the GPS based on lifetime experiences, the researchers conducted a second GPS interview within 24 hours to ensure that all answers corresponded to the symptoms experienced within the previous 30 days.

### Continuation of Therapy

None of the participants requested referral for the continuation of mental health care. Many participants expressed an interest in downloading the app to continue the intervention tasks when it became available.

### Adverse Events

No adverse events were reported during the study.

### Additional Results

Additional results for all analyses proposed are being prepared for publication as of May 2022.

## Discussion

### Principal Findings

We anticipate that this proof-of-concept trial will provide evidence of the preliminary efficacy of the proposed intervention in treating trauma and will identify novel cognitive and electrophysiological diagnostic and prognostic markers of clinical and subclinical PTSD. Although the results of this trial are being prepared for publication, the enrollment outcomes were presented in this protocol. On the basis of the sample size estimation, the aim of this study was to recruit 60 participants. However, refugee community members’ interest in joining the trial exceeded the study capacity. Moreover, the dropout rates were unusually low for PTSD clinical trials, with only 3% (1/35) of the PTSD participants dropping out before treatment initiation and none dropping out during or after treatment. In contrast, other intervention studies have indicated dropout rates >20% [73-75].

### Strengths and Limitations

This study design presents strengths and limitations. First, it is essential to acknowledge that our healthy control group, although not presenting PTSD or other psychiatric disorders, was exposed to similar war and conflict-related traumatic events as the PTSD group. The recruitment of nonexposed controls, although most suitable, was not possible considering that all 3 African refugee communities involved in this study migrated to Australia because they were fleeing conflict. Moreover, between both groups, traumatic brain injury, medication washout, and other medical conditions were only self-reported via the approved clinical interviews. Thus, it is possible that trauma exposure among controls, along with brain injury, unreported use of medications, or other undetected medical conditions between both groups, could affect response to the intervention, cognitive test scores, and quantitative EEG recordings. Particularly in the PTSD group, quantitative EEG recordings can also be affected by the dysregulation of sleep often present in traumatic stress; therefore, this must be considered during the analyses.

Although one of the strengths of this study is the combination of objective and subjective diagnostic and prognostic measures, owing to restricted resources, the follow-up phase did not include the collection of quantitative EEG, biometric data, or cognitive tests. In addition, although the proposed design does not include randomization of participants and a no-intervention group as a proof of concept, this feasibility study serves a vital role in subjectively and objectively examining the preliminary efficacy of the eResilience app before directing resources toward large randomized controlled trials. It is also important to note that, although the proposed app intervention was designed for cross-cultural use, the sample recruited for this study, primarily represents African cohorts exposed to war and conflict-related trauma. Finally, the COVID-19 pandemic coincided with the 12-month follow-up mark, potentially causing additional stress after the intervention.

### Clinical Intervention Design Challenges

To date, apps are unlikely to address all complexities of mental health disorders and, thus far, evidence supports that they are best used to supplement care rather than as first-line interventions. The development of the eResilience app as a high-impact intervention and the proposed study design are challenged by the fact that the populations for which this intervention is designed are likely never to have access to adequate professional help in their lifetime, and an app such as the one proposed may be the only help they will ever access. Moreover, recognizing that PTSD is not the sole mental health concern among our target populations and that the eResilience app should aim toward a transdiagnostic approach, this proposed study lacks the power and resources to examine outcomes across diverse domains. Another considerable challenge relates to designing a tool suitable for large-scale dissemination while not compromising safety and quality of care.

Notably, one of the promising strengths of this intervention design for individual or group use is the cost efficacy projected to be relatively lower than existing programs. In addition, the simplicity and safety of the designed clinical tools aim to eliminate the need for training and supervision of local lay staff. Finally, the week-long time frame would also further reduce costs in extensive community outreach efforts, thus boosting its scalability potential.

### Future Directions

If successful, this trial will provide a foundation for large-scale, cross-cultural studies to further examine the efficacy of the proposed app intervention and biomarker precision as well as to better understand how brain data may inform other cost-effective biometric measures for software integration, serve as a digital aid for the precision diagnosis of PTSD and related disorders, and measure treatment response.

The research software version used in this study is available upon request to all external researchers interested in conducting future trials. Recommendations for future designs include (1) the inclusion of healthy controls that have not been exposed to traumatic stress, particularly as it pertains to the identification of diagnostic markers; (2) the inclusion of objective measures to examine physiological regulation skills at long-term follow-ups; (3) the use of smartphones with sensor technology for biometric data collection, including heart rate variability; (4) the investigation of the efficacy of the app for other disorders as primary outcome measures, such as depression and generalized anxiety; and (5) the expansion of the sample diversity to include non-African communities and demographics affected by other types of humanitarian emergencies besides war and conflict. Finally, although the design and results of this trial may be premature in assessing the cost-efficacy and scalability qualities of the app, we also strongly recommend that those qualities be considered in future study protocols. Further considerations will be discussed alongside the publication of the study results.

### Conclusions

This study protocol introduces a novel digital app for the treatment of posttraumatic stress in emergencies. Its nonreliance on the DSM-5 constructs of PTSD and greater focus on the neural substrates of traumatic stress as an attempt to target the cause of symptoms may increase the likelihood of cross-cultural adaptability, promoting long-term sustainable change on a neurobiological basis rather than via the reduction and ongoing maintenance of symptoms. The potential identification of diagnostic markers could yield further advances for the humanitarian sector, contributing toward the cost-effectiveness of large-scale programs by making the best use of limited resources when identifying persons in need and objectively monitoring impact via automated health data feedback. Overall, we conclude that the successful enrollment and low dropout results presented indicate a strong interest from the Liberian, Sudanese, and Congolese refugee communities in Australia to be involved in research to contribute toward the development of novel approaches to address global mental health challenges caused by war and adversity.

## Appendix

Multimedia Appendix 1 eResilience app research version 1.0.

Multimedia Appendix 2 Demographics for Africans in Australia.

Multimedia Appendix 3 Chronological trauma exposure chart.

Multimedia Appendix 4 App survey for 12-months post-test assessment.

Multimedia Appendix 5 eResilience app response booklet.

Multimedia Appendix 6 Participant information statement.

Multimedia Appendix 7 Participant consent form.

Multimedia Appendix 8 Participant Information Statement - Phase 2.

Multimedia Appendix 9 Participant Consent Form Phase 2.

## Abbreviations

ANS: autonomic nervous system
CAPS-5: Clinician-Administered Posttraumatic Stress Disorder Scale for the Diagnostic and Statistical Manual of Mental Disorders, Fifth Edition
CBT: cognitive behavioral therapy
DSM-IV: Diagnostic and Statistical Manual of Mental Disorders, Fourth Edition
DSM-5: Diagnostic and Statistical Manual of Mental Disorders, Fifth Edition
EEG: electroencephalogram
EGI: Electrical Geodesics
GPS: Global Psychotrauma Screen
HTQ: Harvard Trauma Questionnaire
PM+: Problem Management Plus
PTSD: posttraumatic stress disorder
