# Supplementary material for: Advancing Posttraumatic Stress Disorder Diagnosis and the Treatment of Trauma in Humanitarian Emergencies via Mobile Health: Protocol for a Proof-of-Concept Nonrandomized Controlled Trial
Source: JMIR Res Protoc. 2022 Jun 15;11(6):e38223. doi: 10.2196/38223 (PMC9244657; doi:10.2196/38223)
Supplement: Multimedia Appendix 1 [file resprot_v11i6e38223_app1.pdf]

eResilience App Research Version 1.0 (eRes rv1.0)

Software Content Structure and Wireframes

Content Structure

Section		Title	Frequency	Length
Start Day	I	Welcome	Daily	20’’
Set-Up	II	Set-Up	Days 1 and 2	2’
Scale	III	Wellbeing Scale 1	Daily	10’’
Scale	IV	SUDS 1	Daily	20’’
°C	V	Temperature Check 1	Daily	20’’
Scale	VI	Safety Check 1	Daily	20’’
Block 1 Clinical Tasks			Daily	24m
Scale	VI	Safety Check 2	Daily	20’’
°C	V	Temperature Check 3	Daily	20’’
Block 2 Clinical Tasks			Daily	5-10m
Scale	VI	Safety Check 3	Daily	20’’
°C	V	Temperature Check 4	Daily	20’’
Block 3 Clinical Tasks			Daily	10m
Scale	VI	Safety Check 4	Daily	20’’
°C	V	Temperature Check 5	Daily	20’’
Block 4 Clinical Tasks			Daily	5m
Scale	VI	Safety Check 5	Daily	20’’
°C	V	Temperature Check 6	Daily	20’’
Block 5 Clinical Tasks			Daily	5m
Scale	VI	Safety Check 6	Daily	20’’
°C	V	Temperature Check 7	Daily	20’’
Block 6 Clinical Tasks			Daily	7m
Scale	VI	Safety Check 7	Daily	20’’
°C	V	Temperature Check 8	Daily	20’’
Block 7 Clinical Tasks			Daily	15m
Notification	VII	Daily Completion Instructions	Days 1-6	20’’
Notification		Final Completion	Day 7	20’’
Scale	III	Wellbeing Scale 2	Daily	10’’
Scale	IV	SUDS 2	Daily	20’’
Scale	VI	Safety Check 8	Daily	20’’
°C	V	Temperature Check 9	Daily	20’’

Wireframes

Researcher Dashboard

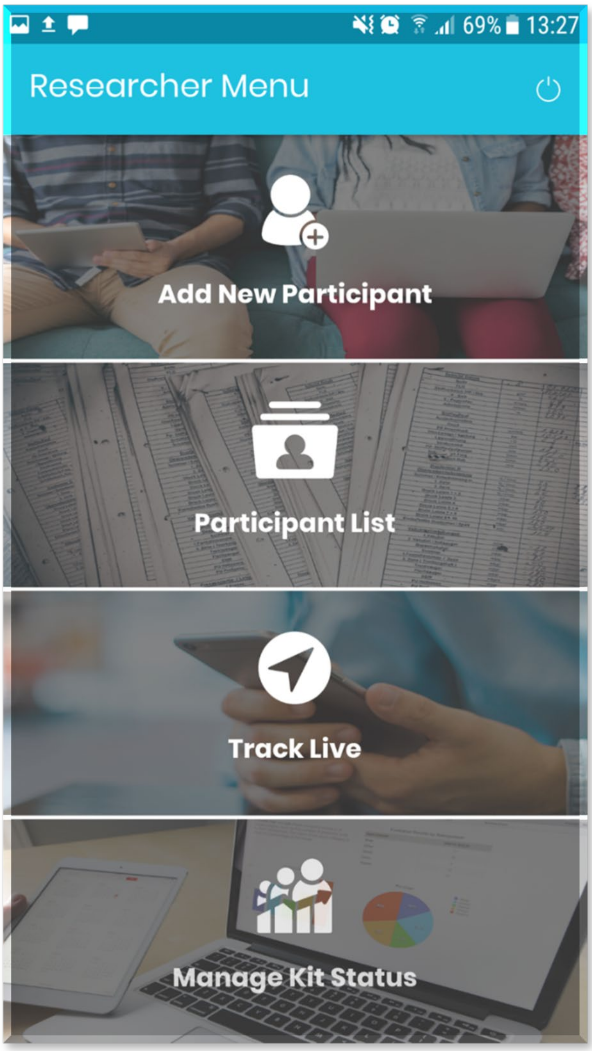

The App opens to the researcher dashboard, presenting four core activities: adding a new participant, access to the participant list, tracking a participant in real time (Track Live) during the intervention, and managing the status for the study kits, which includes the smartphone and biofeedback equipment that participants took home for the seven days.

Add participant

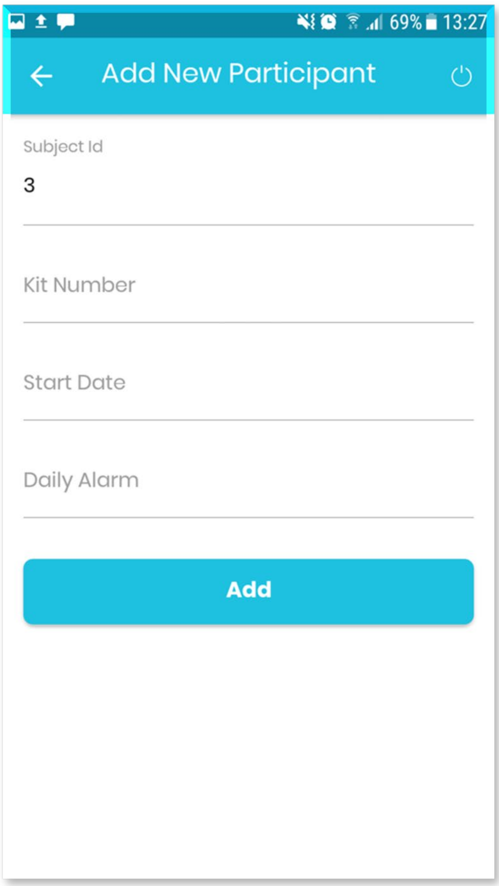

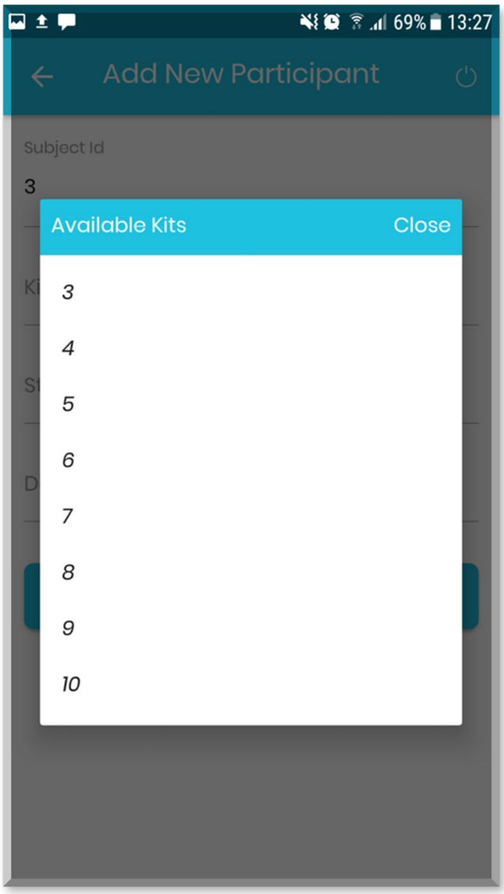

To add a new participant, it was required to input the participant study ID number. To ensure the confidentiality of each individual was protected, this field only permitted a number of inputs. The participant's name was not listed in any part of the software.

When adding a new participant, the researcher can also choose which kit will be assigned to the participant based on availability. In this frame, the App automatically displays only the free kits currently in the lab.

Add participant (continued)

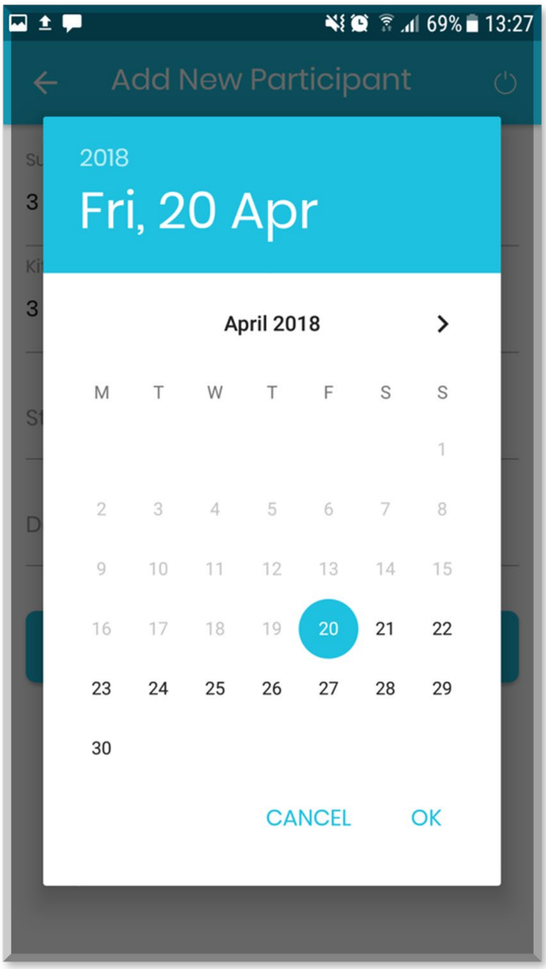

Start Day

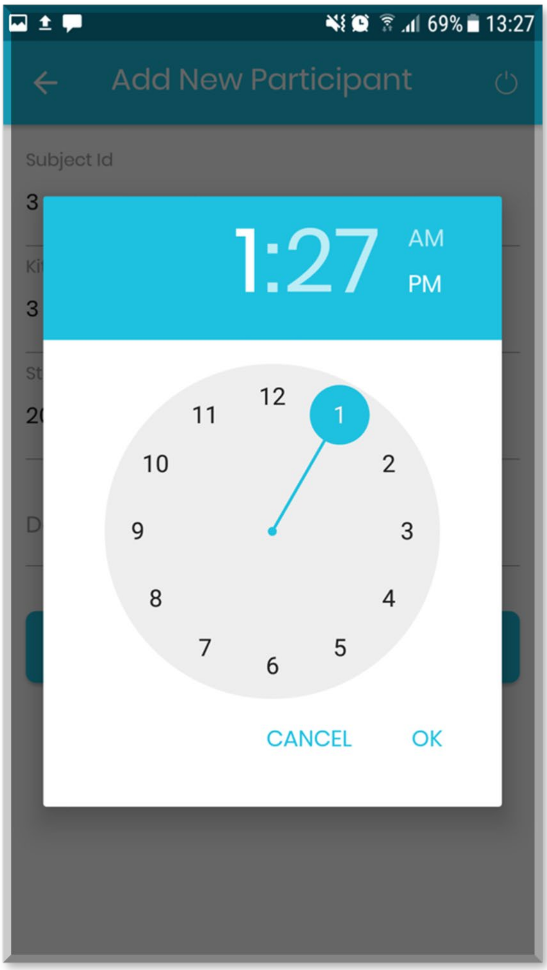

Daily Alarm

The start date corresponds to the day the participant is scheduled to complete day one of the intervention.

It is recommended that the daily alarm feature is determined with the participant to choose which time of the day they wish to complete the intervention daily. Once the alarm is set, it reminds the participant twice daily at 1 hour and then at 15 minutes before the starting time.

Participant List

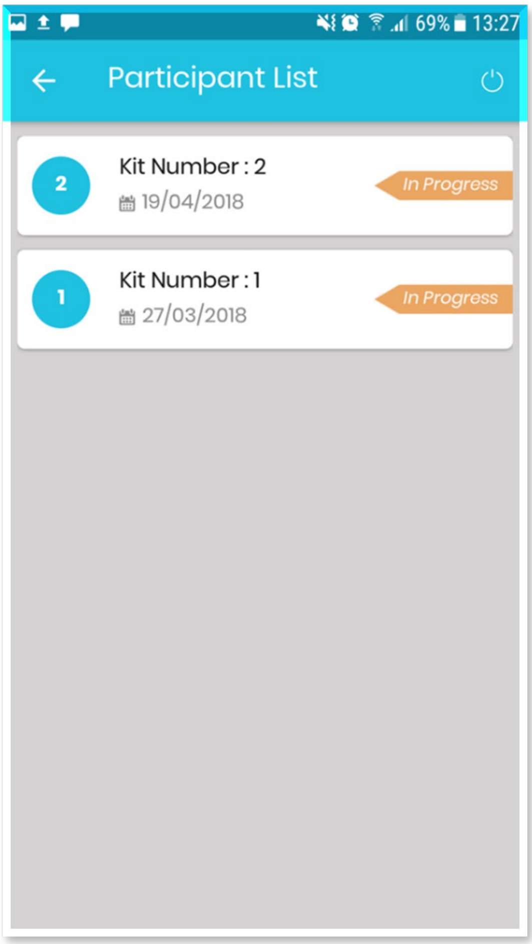

Participant List

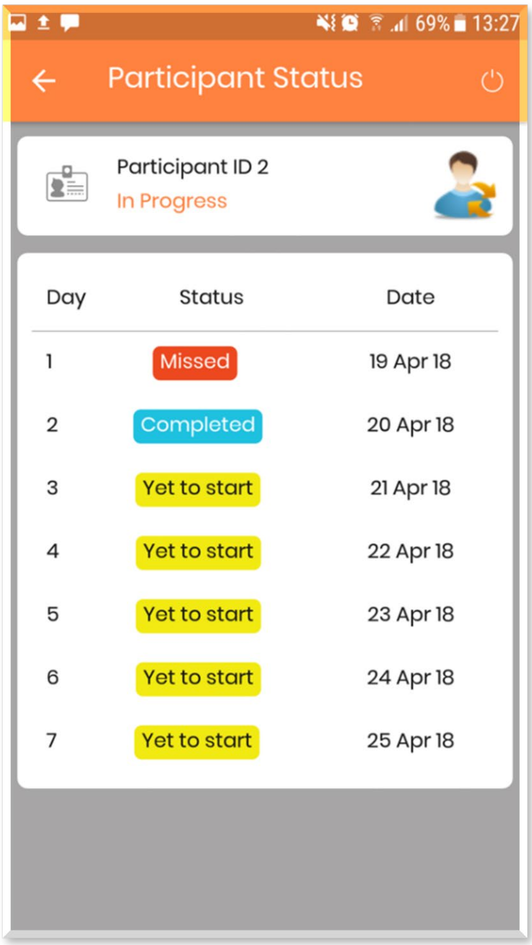

Participant Status

Under the participant list tab, the study ID is displayed in the blue circle, and the researcher has a summary view for each participant status indicated by a red flag (intervention not started), orange flag (intervention in progress), or green flag (intervention complete). Furthermore, by clicking on the participant, the researcher has access to a detailed status of participation, which includes all seven days of the intervention and which days were completed, missed or are yet to start, alongside corresponding dates.

Track Live

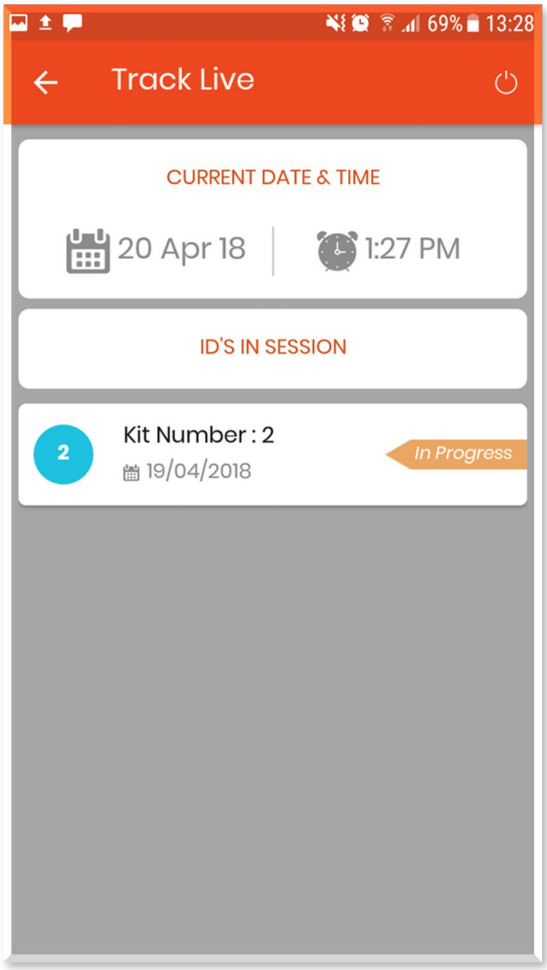

The *Track Live* tab displays in real time all IDs who are currently in session.

Manage Kit Status

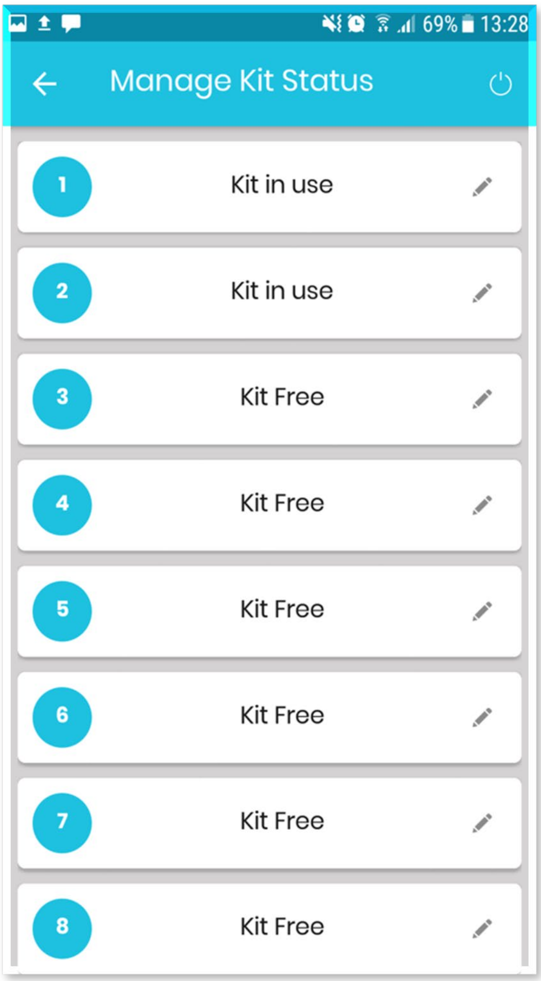

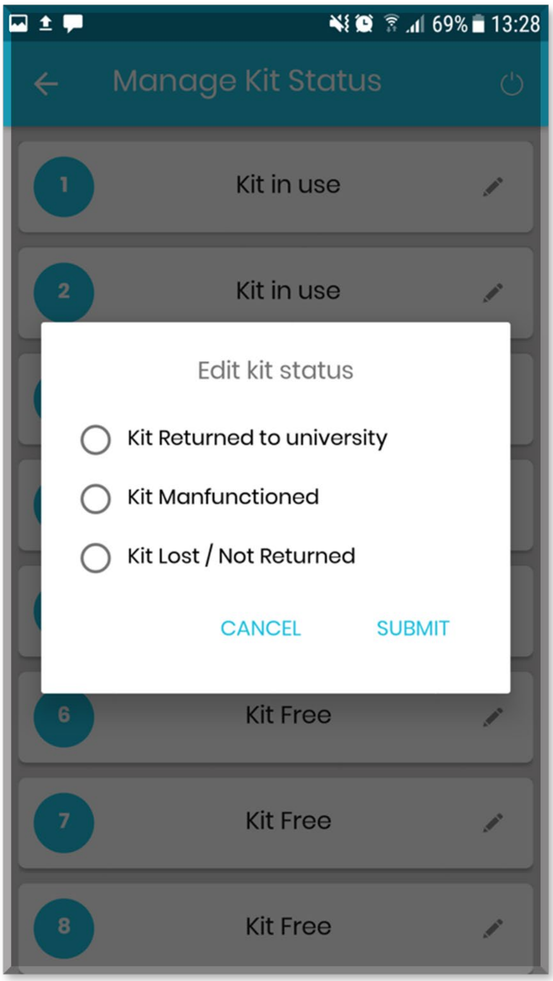

The *Manage Kit Status* tab allows researchers to update the status of each kit, including if the kit has been returned to the university, has malfunctioned, or if it was lost or not returned. The data in this section is reflected only in displaying available kits when assigning one to a new participant.

Participant Dashboard

Daily Participant Welcome Screen

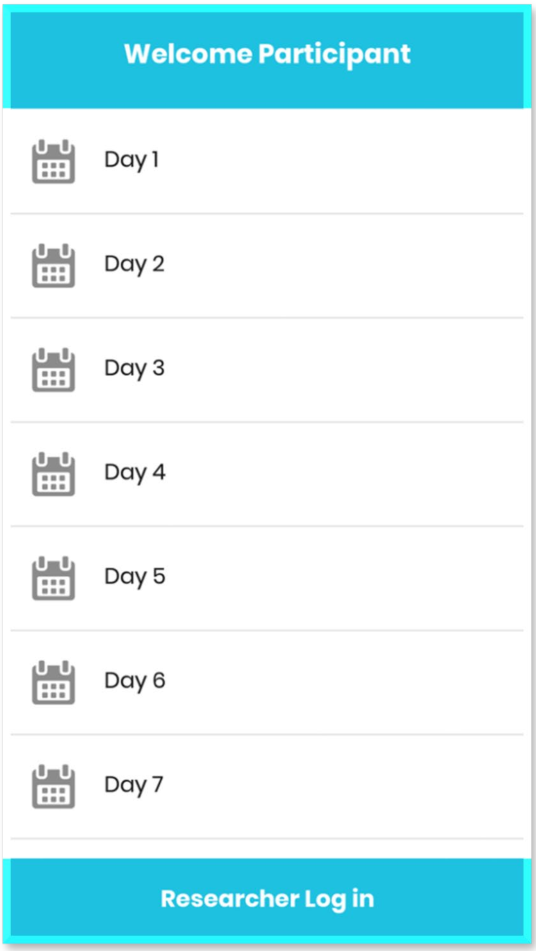

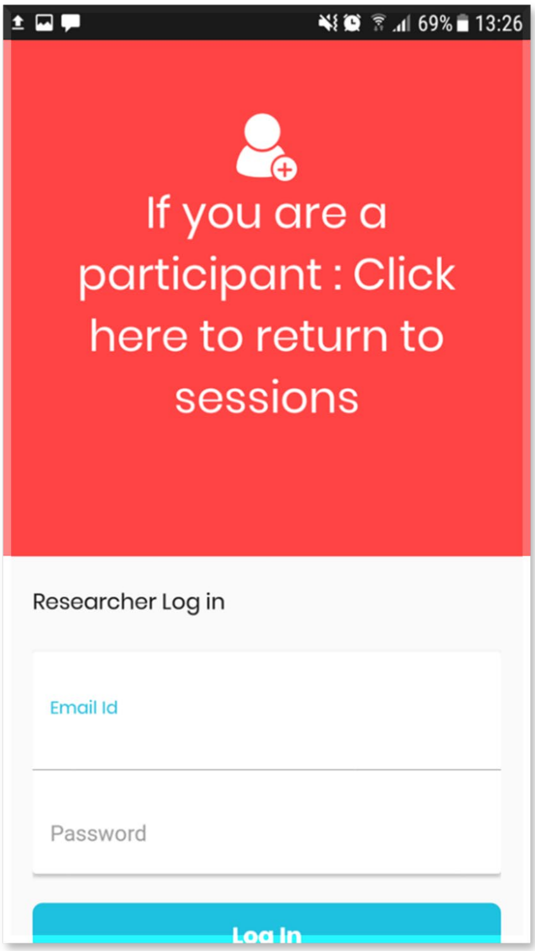

The participant dashboard on the eRes rv1.0 resembles the MVP design, with a simple and intuitive display of each of the seven days of the intervention. In the research version, however, participants could only access each day's activities for 24 hours. If a day was missed, the participant was unable to return and complete it later.

Once the researcher added a new participant and launched the participant dashboard, it was only possible to return to the researcher dashboard via a secure log-in pathway. If participants clicked on the log-in button, they were instructed to return to the participant dashboard.

Daily Biofeedback Set-Up

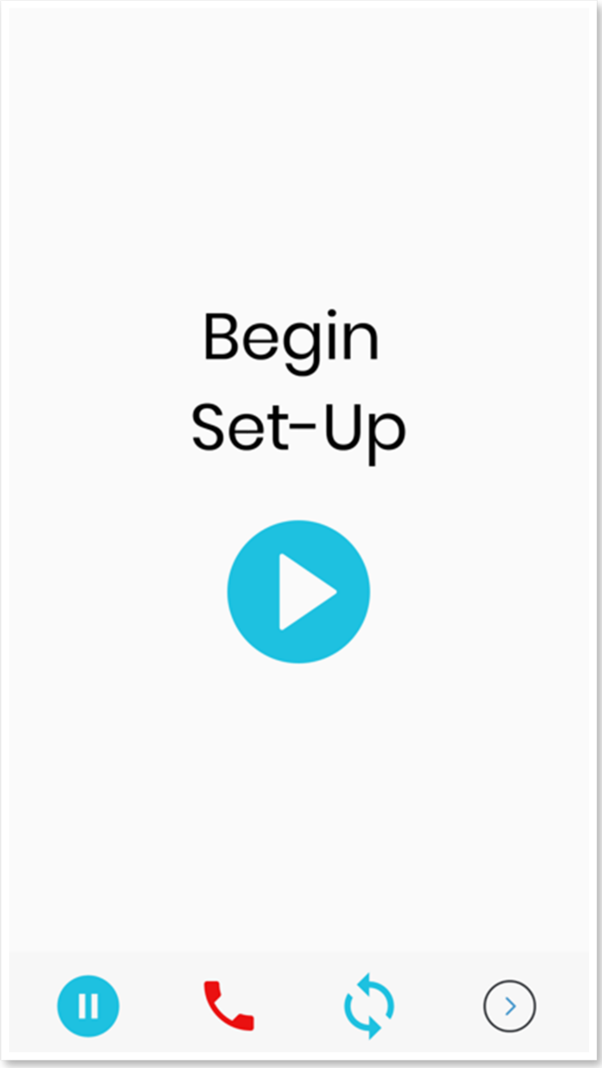

During the first two days, the App guides participants on setting up the App volume, headphones, and biofeedback equipment, which alongside in-person instructions recommended at baseline, and printed materials in the kit, teach individuals how to complete the daily set-up.

Volume Check

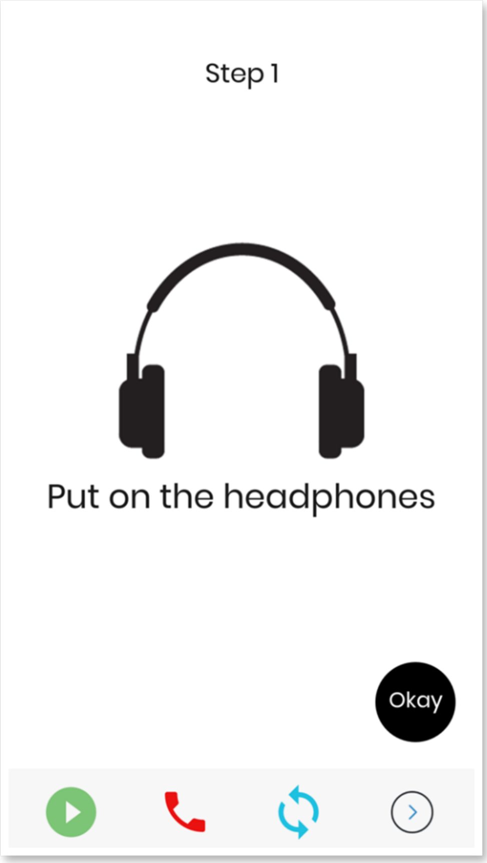

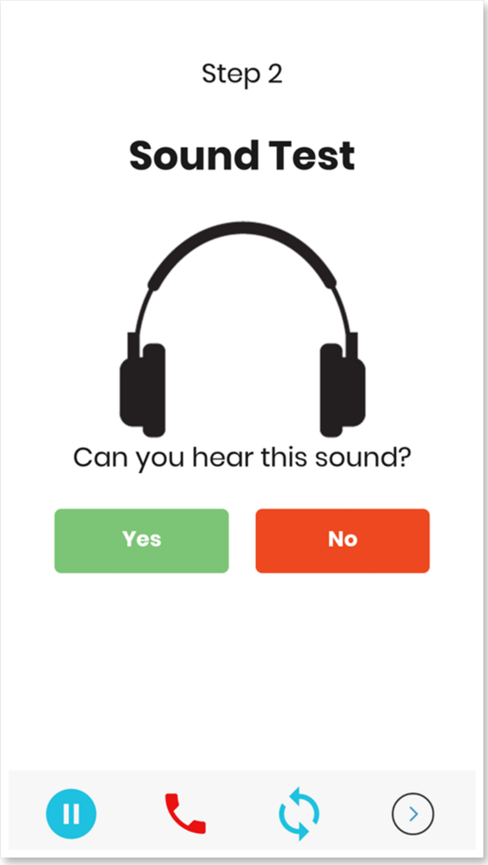

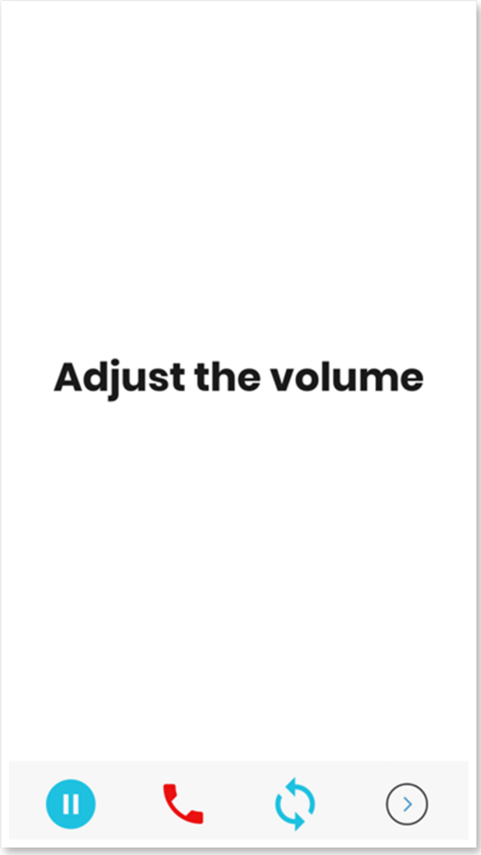

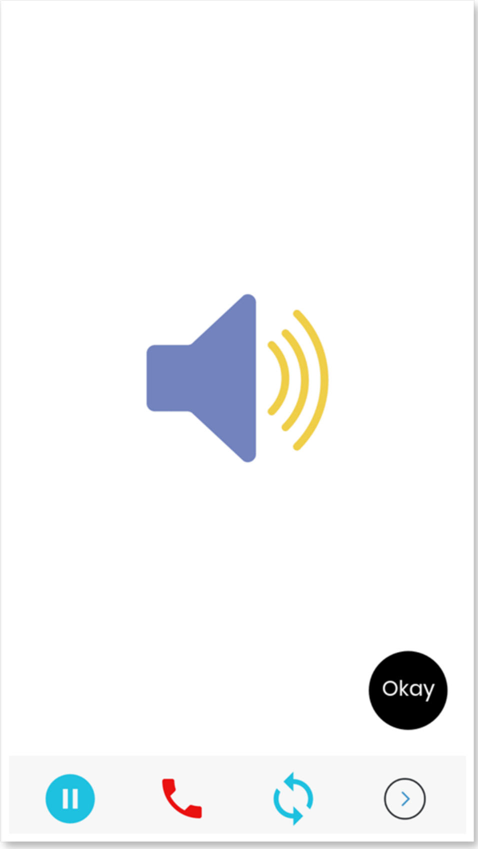

Biofeedback Set-Up

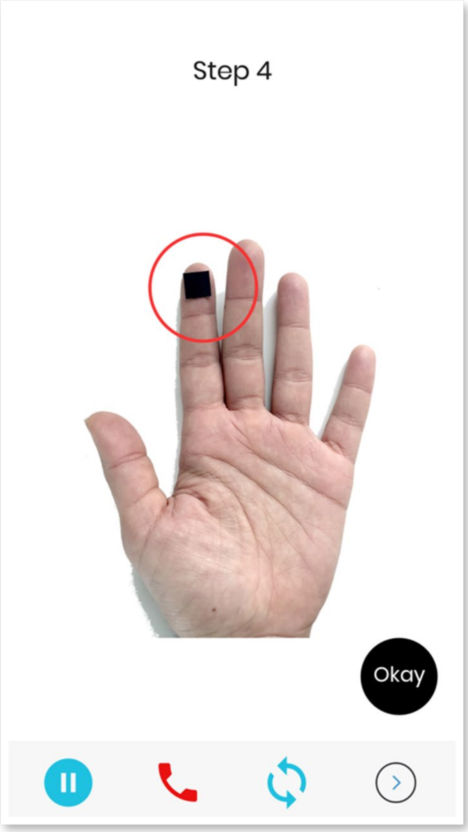

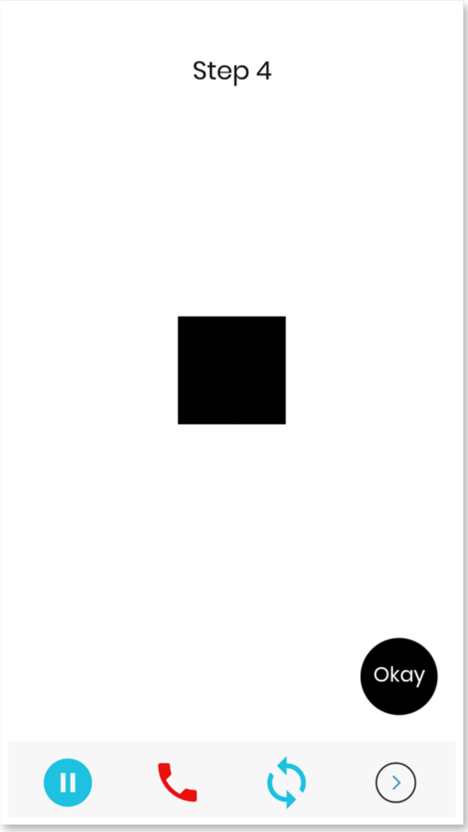

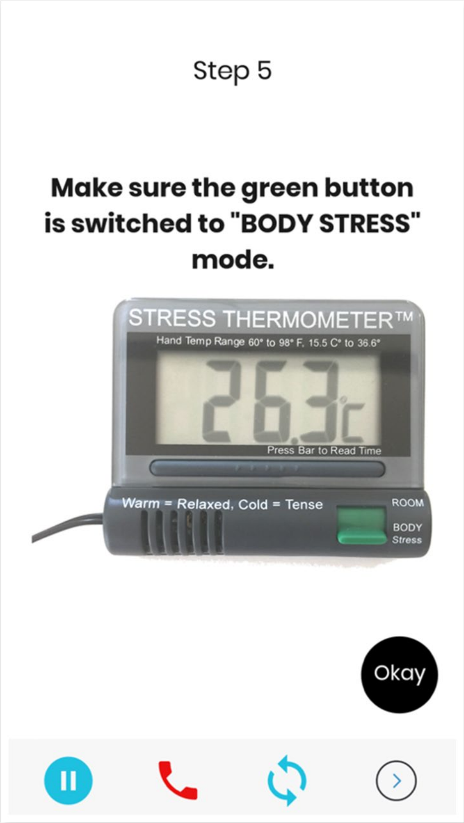

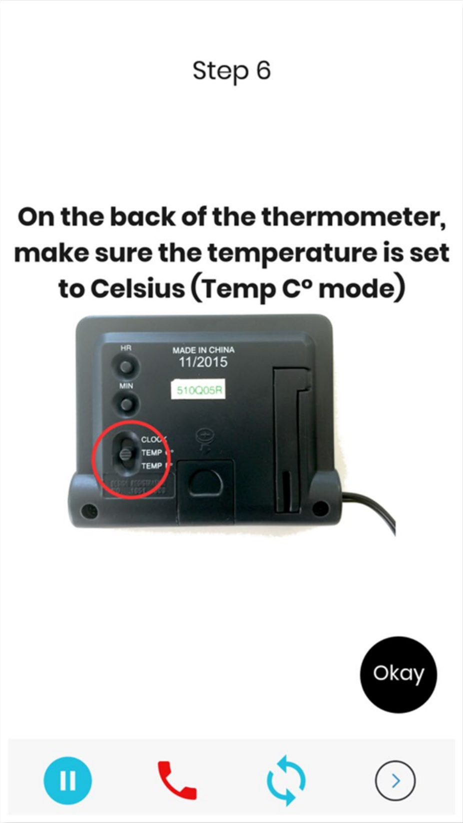

Step 7

Take the blue tip of the thermometer wire to your middle finger tip.

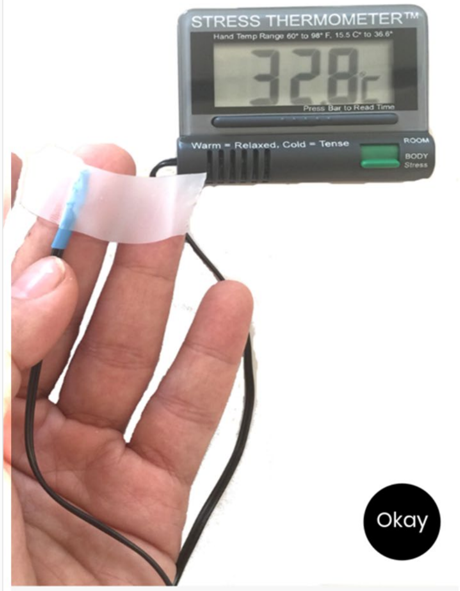

Okay

Are both the biodot sticker and thermometer setup on same hand?

Yes

No

Click Ok when your setup is done

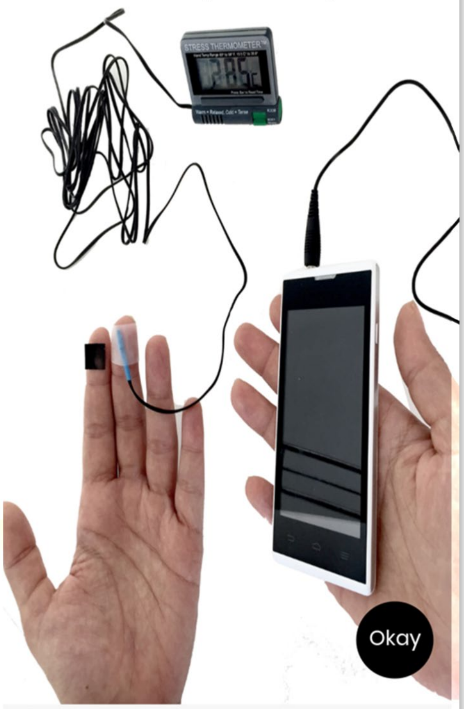

Okay

Reminder

Keep this set-up on for the next 90 minutes until you complete all of todays tasks.

Okay

Daily Check-Ins

The App collects additional daily data at the beginning, end, and between the seven blocks of clinical tasks.

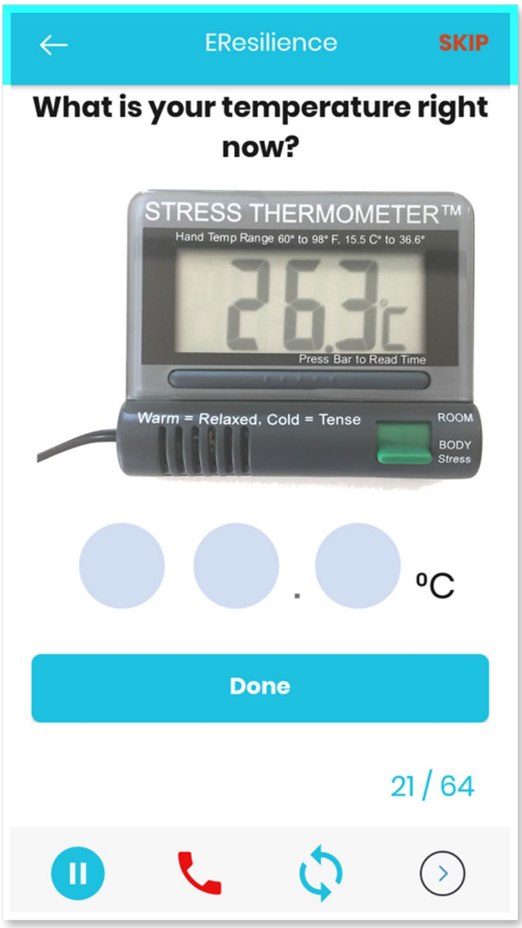

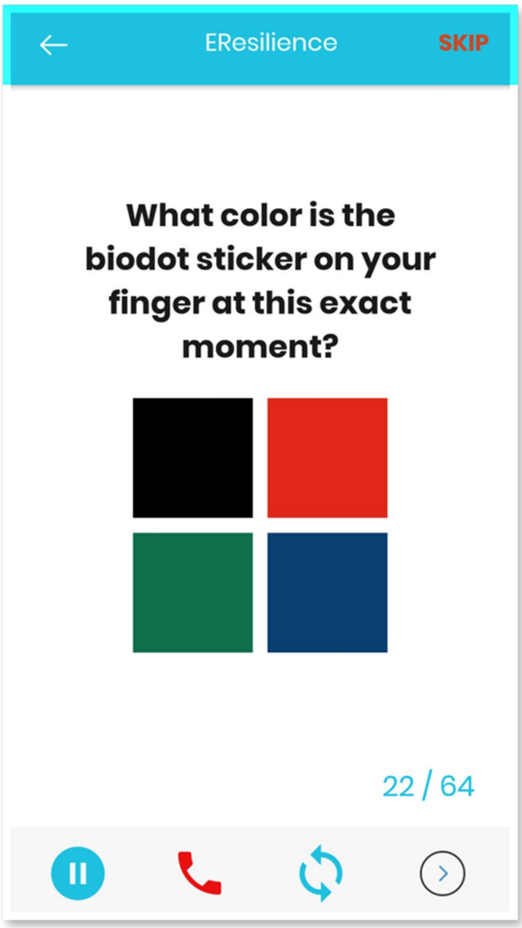

Temperature Checks

The eRes rv1.0 was designed for mid-budget smartphones which do not possess biometric data sensors. Alongside the smartphone, a portable, non-invasive finger temperature thermometer and biodot stickers are provided to participants at home to log-in body temperature changes. The thermometer tip and the Biodot sticker are taped to the fingertips of the hand that is not being utilised to hold the smartphone to prevent the phone from heating the equipment and interfering with the temperature accuracy.

Wellbeing Scale

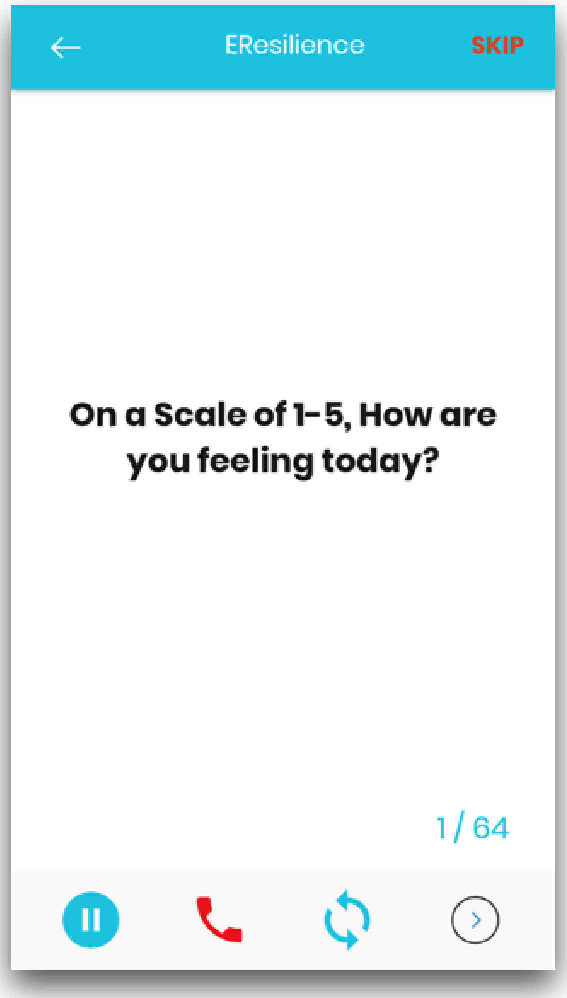

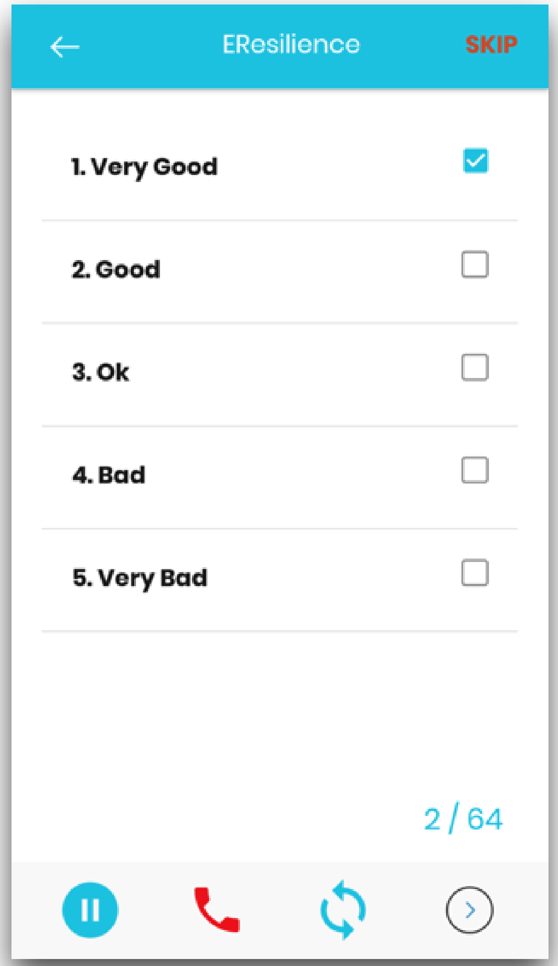

Participants are asked to complete a wellbeing scale. Answers ranged between 1 and 5, with one being feeling very good and five feeling very bad. When a participant answers 4 (bad) or 5 (very bad), the researcher automatically receives a text message notification to call the participant and check their well-being.

Safety Check: Body Discomfort

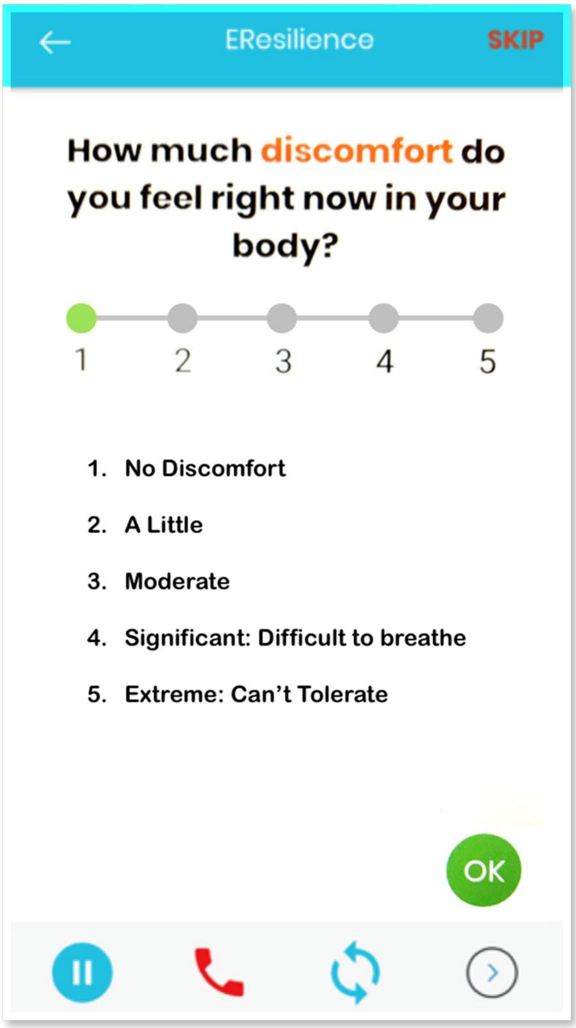

Due to the growing evidence of accentuated somatic complaints related to PTSD and the App's significant content of body-related clinical tools, the App presents a subjective measure of body discomfort at the start and end of each day as a safety measure. Participants answer their level of body discomfort on a scale between 1 and 5, with one being no discomfort and 5 being extreme discomfort. As with the wellbeing scale, if a participant answer 4 (significant discomfort) or 5, the researcher is notified and instructed to contact the participant.

Subjective Units of Distress Scale (SUDS)

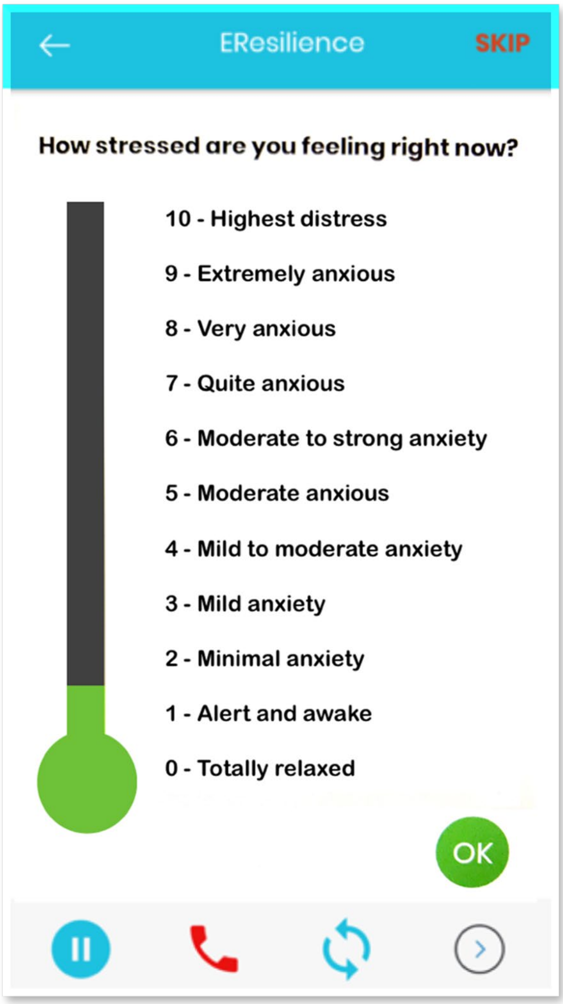

The Subjective Units of Distress Scale (SUDS) is presented at the start and end of each day to measure self-reported stress levels. Participants input responses from a scale of 1-10, with one being totally relaxed and ten being the highest stress level.

Clinical Tasks

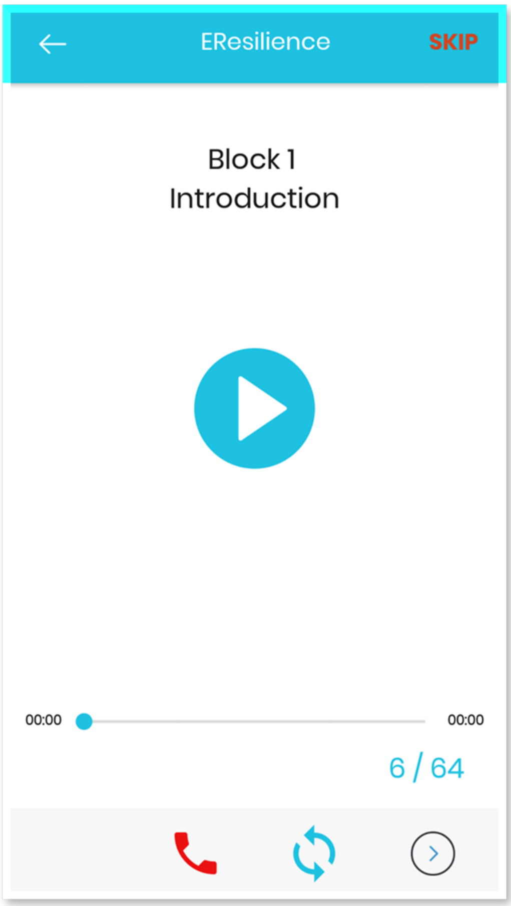

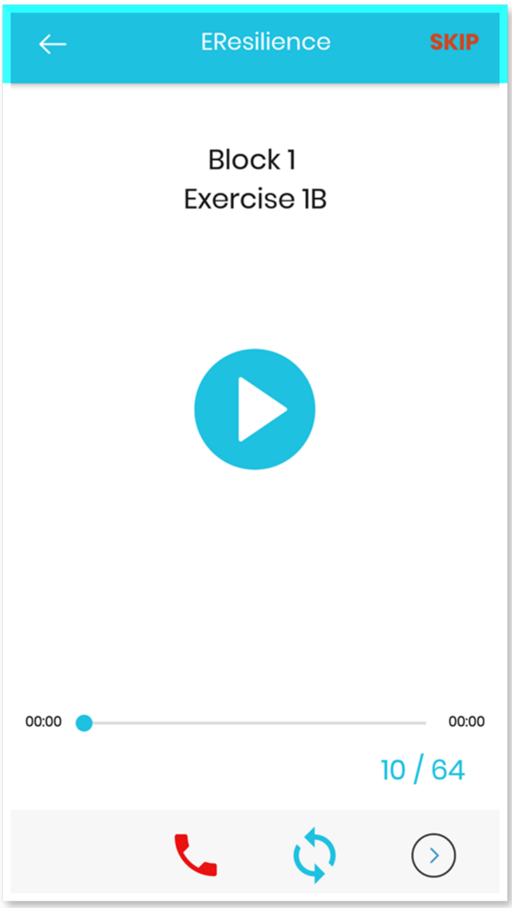

The interface for the clinical tools presents a straightforward and intuitive layout, with a play and pause button to listen to and complete each task. Although the timestamp is displayed, participants are unable to fast-forward the tracks.

End of Day Data Submission and Support

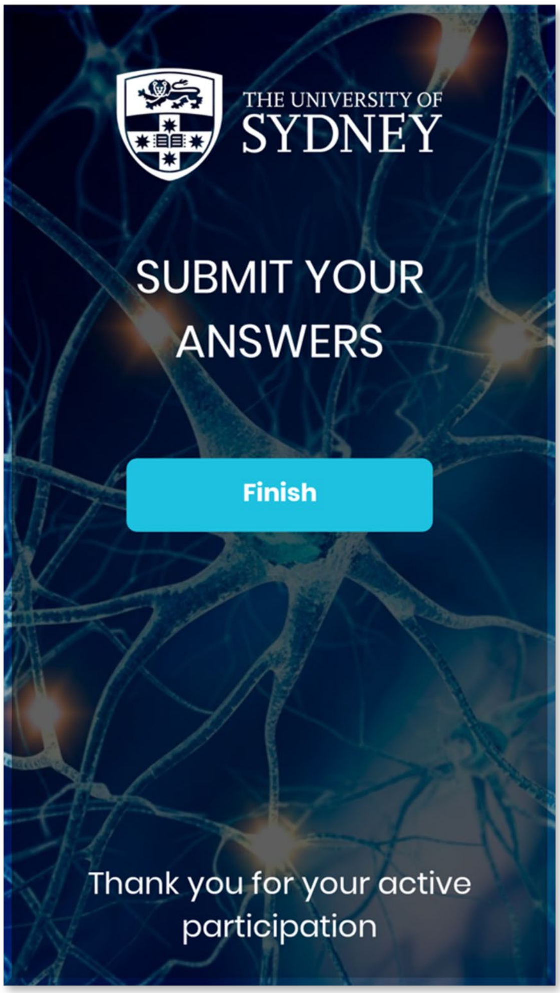

At the end of each day, participants are notified when they have concluded all tasks and are asked to submit their answers.

Additional Safety Measures

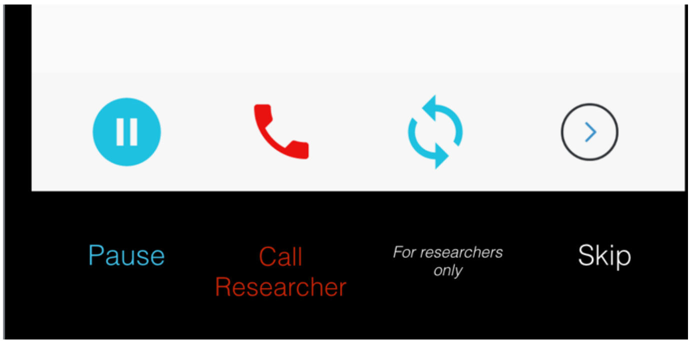

A telephone icon is always present, allowing participants to call the researcher at any time.

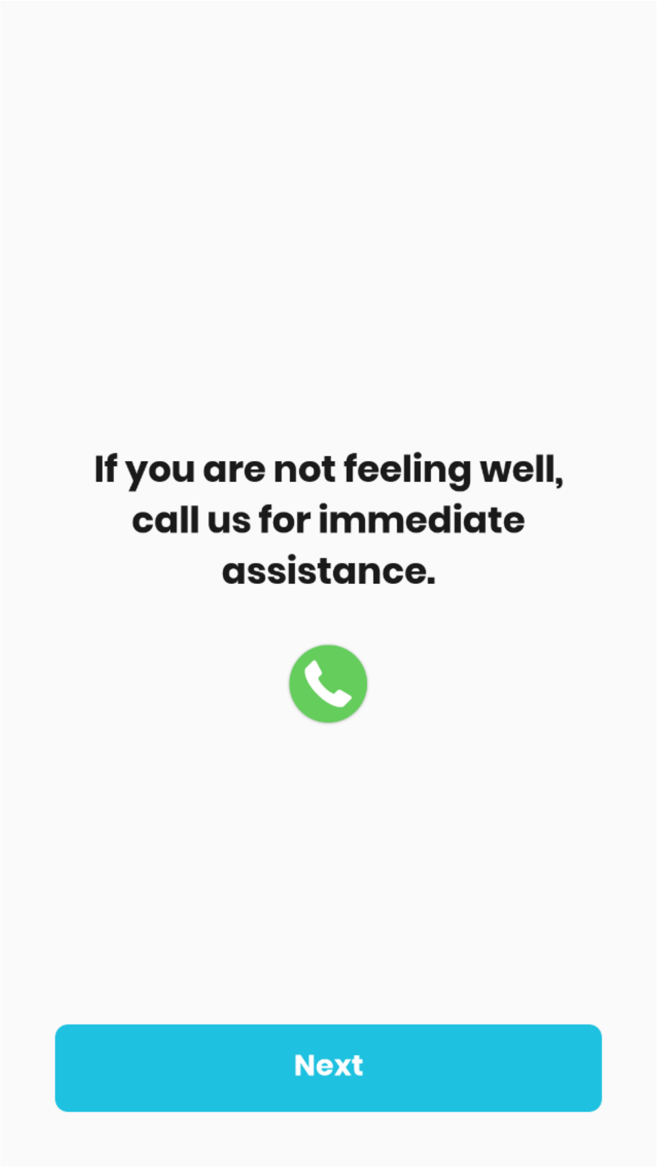

Moreover, the App prompts participants to call the researchers if they feel unwell and require immediate assistance at the end of each day.
