# Supplementary material for: Advancing Posttraumatic Stress Disorder Diagnosis and the Treatment of Trauma in Humanitarian Emergencies via Mobile Health: Protocol for a Proof-of-Concept Nonrandomized Controlled Trial
Source: JMIR Res Protoc. 2022 Jun 15;11(6):e38223. doi: 10.2196/38223 (PMC9244657; doi:10.2196/38223)
Supplement: Multimedia Appendix 2 [file resprot_v11i6e38223_app2.pdf]

DEMOGRAPHICS FOR AFRICANS IN AUSTRALIA

We would like to learn some general information about you.

DATE OF BIRTH

Day: Month: Year:

ETHNIC BACKGROUND

(circle one) LIBERIAN SUDANESE CONGOLESE

1. In which region or *county (region in your country)* were you born in?

2. Do you have an ethnic group?

LANGUAGE

3. Do you have a native dialect? ( ) Yes ( ) No

4. How many/which languages and dialects you speak?

5. How long have you been speaking English?

6. How Difficult is it for you to Understand English?
( ) Very Difficult
( ) A little Difficult
( ) Not Difficult

7. How Difficult it is to Communicate in English?
( ) Very Difficult
( ) A little Difficult
( ) Not Difficult

7. Can you Read American English? ( ) Yes ( ) No

FAMILY BACKGROUND

9. What is your marital status?	___ Single<br>___ Engaged<br>___ Married #spouses___<br>___ Separated<br>___ Divorced<br>___ Widowed<br>[if Y, due to war? Y N]
If currently married, where is your spouse now?	___ Africa ___AUS<br>___Other ___N/A
2. Do you have children?	___Y ___N
If you have children, how many and where are them now?	___ Africa ___AUS<br>___Other ___N/A<br>___Deceased<br>*if yes*, due to war Y N<br>Ebola Y N

MIGRATION AND EXILE

10. What year did you arrive in Aus?	
11. How did you arrive?	
12. Who did you come with?	
13. How many family members are living with you in Australia?	
14. How many people do you presently live with?	
15. Were you born during war?	Y N
16. Were born in a refugee camp?	Y N
17. Did you live in refugee camps?	Y N
18. *If yes*, for how long and how old were you?	

WORK & EDUCATION HISTORY

19. What was your occupation in Africa?	
20. What is your current employment or source of income?	
21. What is the highest level of Education you have completed?	

MENTAL HEALTH HISTORY

22. Were you referred to receive mental health support when you arrived in Australia?	Y N
23. Have you seen a psychologist or psychiatrist for trauma or other mental health support?	Y N
if yes: 24. how many sessions?	
if yes: 25. Did it help you?	
26. Have you ever been diagnosed with a mental health disorder? (if yes, write down details)	

27. Are you right or left handed?	Right	Left
