# Supplementary material for: Advancing Posttraumatic Stress Disorder Diagnosis and the Treatment of Trauma in Humanitarian Emergencies via Mobile Health: Protocol for a Proof-of-Concept Nonrandomized Controlled Trial
Source: JMIR Res Protoc. 2022 Jun 15;11(6):e38223. doi: 10.2196/38223 (PMC9244657; doi:10.2196/38223)
Supplement: Multimedia Appendix 3 [file resprot_v11i6e38223_app3.pdf]

Chronological Trauma Exposure Chart (CTEC) v2: Liberia, Sudan and DR Congo

			Year of Birth:	2000	1999	1998	1997	1996	1995	1994	1993	1992	1991	1990	1989	1988	1987	1986	1985	1984	1983	1982	1981	1980	1979	1978	1977	1976	1975	1974	1973	1972	1971	1970	1969	1968
**Years**	**Congo**	**Liberia**	**Sudan**																																	
1989		☐ 1st War	☐ 2nd War													1	2	3	4	5	6	7	8	9	10	11	12	13	14	15	16	17	18	19	20	21
1990															1	2	3	4	5	6	7	8	9	10	11	12	13	14	15	16	17	18	19	20	21	22
1991														1	2	3	4	5	6	7	8	9	10	11	12	13	14	15	16	17	18	19	20	21	22	23
1992													1	2	3	4	5	6	7	8	9	10	11	12	13	14	15	16	17	18	19	20	21	22	23	24
1993												1	2	3	4	5	6	7	8	9	10	11	12	13	14	15	16	17	18	19	20	21	22	23	24	25
1994											1	2	3	4	5	6	7	8	9	10	11	12	13	14	15	16	17	18	19	20	21	22	23	24	25	26
1995										1	2	3	4	5	6	7	8	9	10	11	12	13	14	15	16	17	18	19	20	21	22	23	24	25	26	27
1996									1	2	3	4	5	6	7	8	9	10	11	12	13	14	15	16	17	18	19	20	21	22	23	24	25	26	27	28
1997		☐ Fragile peace						1	2	3	4	5	6	7	8	9	10	11	12	13	14	15	16	17	18	19	20	21	22	23	24	25	26	27	28	29
1998							1	2	3	4	5	6	7	8	9	10	11	12	13	14	15	16	17	18	19	20	21	22	23	24	25	26	27	28	29	30
1999		☐ 2nd War				1	2	3	4	5	6	7	8	9	10	11	12	13	14	15	16	17	18	19	20	21	22	23	24	25	26	27	28	29	30	31
2000					1	2	3	4	5	6	7	8	9	10	11	12	13	14	15	16	17	18	19	20	21	22	23	24	25	26	27	28	29	30	31	32
2001				1	2	3	4	5	6	7	8	9	10	11	12	13	14	15	16	17	18	19	20	21	22	23	24	25	26	27	28	29	30	31	32	33
2002				2	3	4	5	6	7	8	9	10	11	12	13	14	15	16	17	18	19	20	21	22	23	24	25	26	27	28	29	30	31	32	33	34
2003				3	4	5	6	7	8	9	10	11	12	13	14	15	16	17	18	19	20	21	22	23	24	25	26	27	28	29	30	31	32	33	34	35
2004		☐ Fragile peace; Repa-triation		4	5	6	7	8	9	10	11	12	13	14	15	16	17	18	19	20	21	22	23	24	25	26	27	28	29	30	31	32	33	34	35	36
2005			☐ Peace Agreement	5	6	7	8	9	10	11	12	13	14	15	16	17	18	19	20	21	22	23	24	25	26	27	28	29	30	31	32	33	34	35	36	37
2006			☐ Fragile peace	6	7	8	9	10	11	12	13	14	15	16	17	18	19	20	21	22	23	24	25	26	27	28	29	30	31	32	33	34	35	36	37	38
2007				7	8	9	10	11	12	13	14	15	16	17	18	19	20	21	22	23	24	25	26	27	28	29	30	31	32	33	34	35	36	37	38	39
2008				8	9	10	11	12	13	14	15	16	17	18	19	20	21	22	23	24	25	26	27	28	29	30	31	32	33	34	35	36	37	38	39	40
2009			☐ Independen ce conflicts	9	10	11	12	13	14	15	16	17	18	19	20	21	22	23	24	25	26	27	28	29	30	31	32	33	34	35	36	37	38	39	40	41
2010				10	11	12	13	14	15	16	17	18	19	20	21	22	23	24	25	26	27	28	29	30	31	32	33	34	35	36	37	38	39	40	41	42
2011				11	12	13	14	15	16	17	18	19	20	21	22	23	24	25	26	27	28	29	30	31	32	33	34	35	36	37	38	39	40	41	42	43
2012			☐ State conflicts	12	13	14	15	16	17	18	19	20	21	22	23	24	25	26	27	28	29	30	31	32	33	34	35	36	37	38	39	40	41	42	43	44
2013		☐ Ebola Virus	☐ Civil War	13	14	15	16	17	18	19	20	21	22	23	24	25	26	27	28	29	30	31	32	33	34	35	36	37	38	39	40	41	42	43	44	45
2014				14	15	16	17	18	19	20	21	22	23	24	25	26	27	28	29	30	31	32	33	34	35	36	37	38	39	40	41	42	43	44	45	46
2015			☐ Peace talks	15	16	17	18	19	20	21	22	23	24	25	26	27	28	29	30	31	32	33	34	35	36	37	38	39	40	41	42	43	44	45	46	47
2016		☐ Peace Period	☐ Civil war Ethnic Cleansing Famine	16	17	18	19	20	21	22	23	24	25	26	27	28	29	30	31	32	33	34	35	36	37	38	39	40	41	42	43	44	45	46	47	48
2017				17	18	19	20	21	22	23	24	25	26	27	28	29	30	31	32	33	34	35	36	37	38	39	40	41	42	43	44	45	46	47	48	49
2018				18	19	20	21	22	23	24	25	26	27	28	29	30	31	32	33	34	35	36	37	38	39	40	41	42	43	44	45	46	47	48	49	50
**2019**				19	20	21	22	23	24	25	26	27	28	29	30	31	32	33	34	35	36	37	38	39	40	41	42	43	44	45	46	47	48	49	50	51
				**Current Participant Ages in 2019**																																

Did You Experience Any of the Following War-Related Experiences?

This questionnaire is highly confidential and there are no legal consequences to answering these questions. You may choose not to answer any question that makes you feel uncomfortable.

1. Separation from Family: ☐ **Y** ☐ **N**

*W*ho were you separated from and how old were you?

2. Exile

☐ IDP Camp Ages:________ *Duration*: ________
☐ Refugee Camp Ages:________ *Duration*: ________
☐ I left my home directly to Australia
☐ I wish not to answer this question.

3. Bush Hiding

☐ Home country Ages:________ *Duration*: ________
☐ Foreign country Ages:________ *Duration*: ________
☐ I never needed to hide in the bush for safety.
☐ I wish not to answer this question.

4. Malnourishment

☐ No food access Ages:________ *Duration*: ________
☐ Limited food access Ages:________ *Duration*: ________
☐ I did not struggle with lack of food.
☐ I wish not to answer this question.

5. Sexual Assault or Rape

☐ Several Times *Ages*:________
☐ Few times *Ages*:________
☐ Once *Age*: ________
☐ I never experienced sexual assault or rape.
☐ I wish not to answer this question.

6. Physical Torture

☐ Many Times Ages:________
☐ Few times Ages:________
☐ Once Age: ________
☐ I never experienced physical torture.
☐ I wish not to answer this question.

7. Cannibalism (*Forced to eat human remains*)

☐ Many Times Ages:________
☐ Few times Ages:________
☐ Once Age: ________
☐ I was never forced to canibalism.

8. Fighting in the War
Check all that apply

☐ War combatant Ages:________ *Duration*: ________
☐ Combatant as a Child Ages:________ *Duration*: ________
☐ Forced to Cause Harm to Others for survival Ages:___________
☐ Forced to take someone's life for survival Ages:___________
☐ I never had to fight in the war to survive.
☐ I wish not to answer this question.
