# Supplementary material for: Advancing Posttraumatic Stress Disorder Diagnosis and the Treatment of Trauma in Humanitarian Emergencies via Mobile Health: Protocol for a Proof-of-Concept Nonrandomized Controlled Trial
Source: JMIR Res Protoc. 2022 Jun 15;11(6):e38223. doi: 10.2196/38223 (PMC9244657; doi:10.2196/38223)
Supplement: Multimedia Appendix 4 [file resprot_v11i6e38223_app4.pdf]

App Survey (12-Month Follow-Up)

1. Have you practiced any of the exercises you learned on the app on your own or with friends and family during the past year (between your last visit to the research centre on the Sunshine Coast and now)?

YES

How many times have you practiced the tasks after the study without the App?

- ☐ One time in the past year
- ☐ 2 to 14 times in the past year
- ☐ More than 14 times in the past year

Which Months in the Past year did you do it?

- ☐ Mostly in the first 3 months
- ☐ Mostly in the first 6 months
- ☐ Across the Past 12 months

What Frequency did you do it?

- ☐ Random days without firm frequency
- ☐ Only when I needed to help me feel better
- ☐ I incorporated it in my routine:
  - ☐ Every Month
  - ☐ Every Week
  - ☐ Every Day

NO

Would you have continued to use it if you had a copy of the app on your own phone, and could choose to complete only your favourite exercises?

YES **NO**

At What Moments Would the App Be Helpful to You?

- ☐ When I am stressed
- ☐ When someone I care about is stressed and I can help them
- ☐ When I wake up so I can start the day calm
- ☐ At the end of the day to help me fall asleep
- ☐ Whenever I feel traumatised
- ☐ Whenever I feel sad

2. Have you told other people (friends, family or strangers) about the app and skills you learned?

YES **NO**

To How Many People?

3. Have you taught other people (friends, family or strangers) any tasks you learned on the app?

YES **NO**

To How Many People?

4. Do/Did you have a Favourite Exercise /Task on the App that makes you feel best?

YES **NO** **I Don't Remember**

Which One(s)?

5. Did the Skills you Learned Improved your life?

YES **NO**

How So?

6. What Helped You the Most Throughout Life to Overcome Adversity?

- ☐ **1. Family and Community**
- ☐ **2. Faith (Circle)** Christian, Muslim, Other
- ☐ **3. Mental Health Treatment**
- ☐ **4. Vocation/ Purpose**
- ☐ **5. Other**

7. How Much Did Religion Play a Role in your Strength Adapting to Life after War and/or Difficult Times?

- ☐ **1. No Role**
- ☐ **2. Some Role**
- ☐ **3. Major Role**
