# Supplementary material for: Advancing Posttraumatic Stress Disorder Diagnosis and the Treatment of Trauma in Humanitarian Emergencies via Mobile Health: Protocol for a Proof-of-Concept Nonrandomized Controlled Trial
Source: JMIR Res Protoc. 2022 Jun 15;11(6):e38223. doi: 10.2196/38223 (PMC9244657; doi:10.2196/38223)
Supplement: Multimedia Appendix 5 [file resprot_v11i6e38223_app5.pdf]

App Response Booklet

DAY 1

Exercise 1C

Who did you bring today:

Exercise 2A

Who did you think about:

Exercise 3C

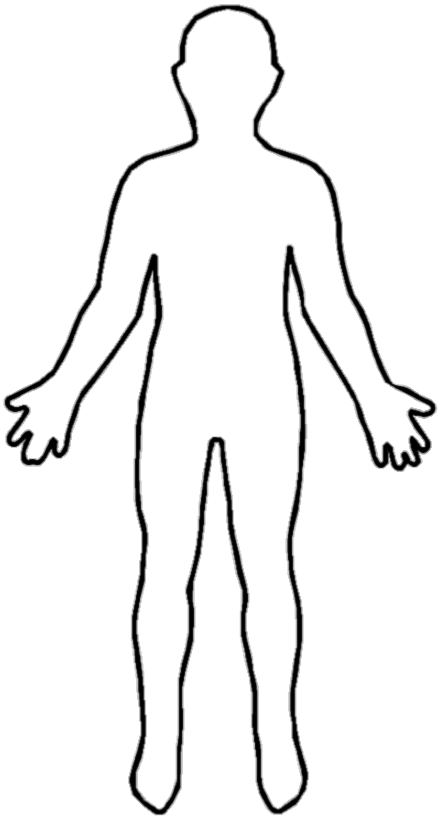

☐ **No discomfort.**

DAY 1

Exercise 5A

Today's Object:

Exercise 5B

5 things
I can see

1:

2:

3:

4:

5:

4 Things
I can touch

1:

2:

3:

4:

3 Things
I can hear

1:

2:

3:

2 things
I can smell

1:

2:

1 thing
I can taste

1:

DAY 2

Exercise 1C

Who did you bring today:

Exercise 3C

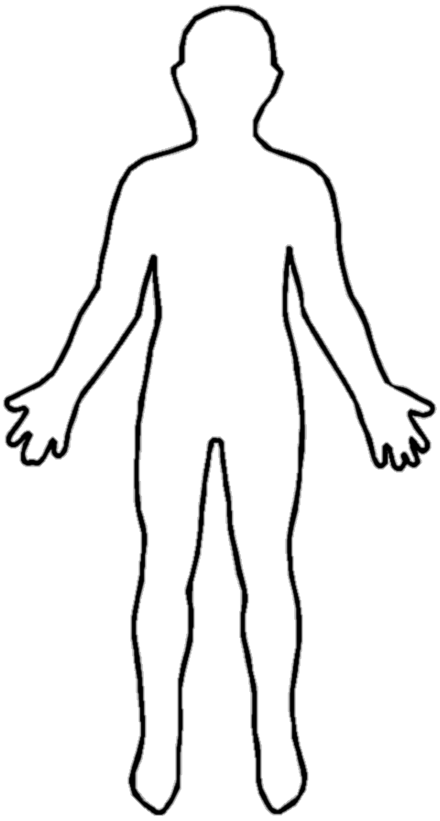

☐ **No discomfort.**

Exercise 5C

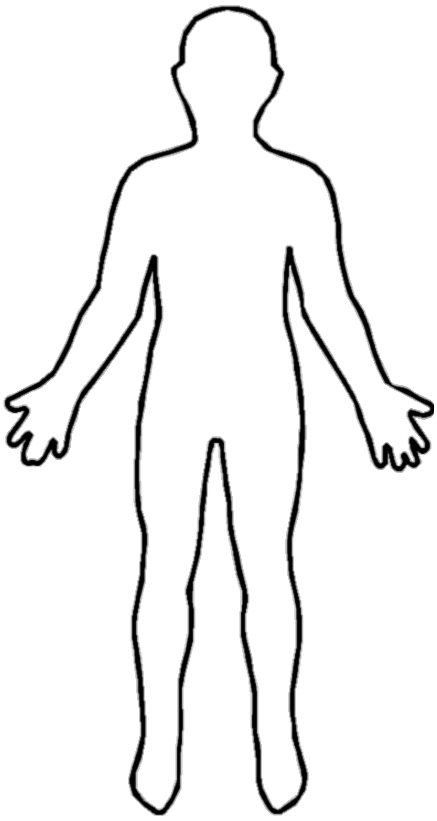

☐ **No discomfort.**

Exercise 7B

Day 2 Altruism Task

Who did you help:

How did you help:

How much time did you spend on this task:

- ☐ Less than 1 hour
- ☐ Between 1-2 hours
- ☐ Between 2-5 hours
- ☐ Half of the day
- ☐ All day

DAY 3

Exercise 1C

Who did you bring today:

Exercise 2B

Nurturing and kind person:

Briefly describe the moment:

Positive Compliment:

Exercise 3C

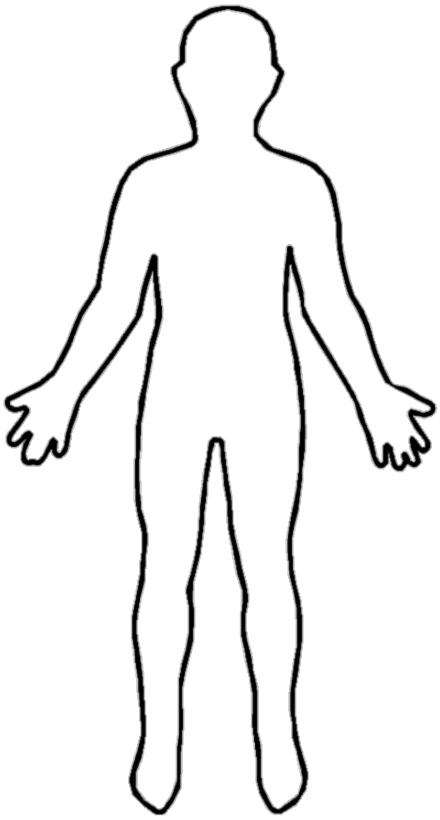

☐ **No discomfort.**

Exercise 7B

Day 3 Altruism Task

Who did you help:

How did you help:

How much time did you spend on this task:

☐ Less than 1 hour

☐ Between 1-2 hours

☐ Between 2-5 hours

☐ Half of the day

☐ All day

DAY 4

Exercise 1C

Who did you bring today:

Exercise 2C

Person 1:

Person 2:

Person 3:

Situation 1:

Situation 2:

Situation 3:

Exercise 3C

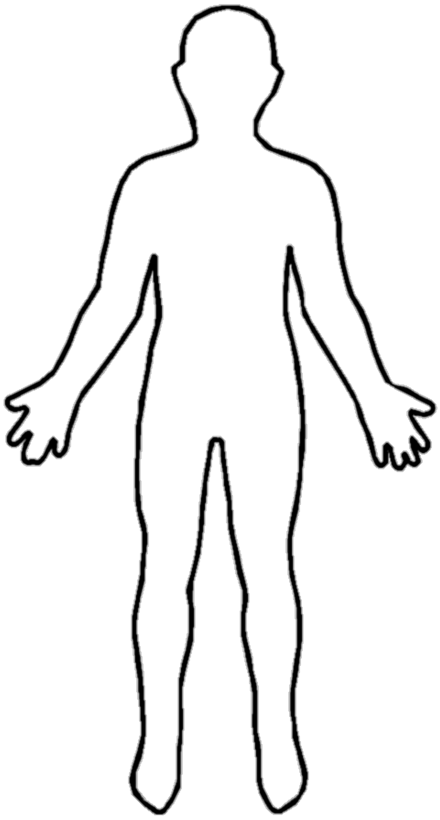

☐ **No discomfort.**

Exercise 5D

Positive List

1.

2.

3.

Exercise 7B

Day 4 Altruism Task

Who did you help:

How did you help:

How much time did you spend on this task:

☐ Less than 1 hour

☐ Between 1-2 hours

☐ Between 2-5 hours

☐ Half of the day

☐ All day

DAY 5

Exercise 1C

Who did you bring today:

Exercise 2D

Situation 1:

Situation 2:

Situation 3:

Exercise 3C

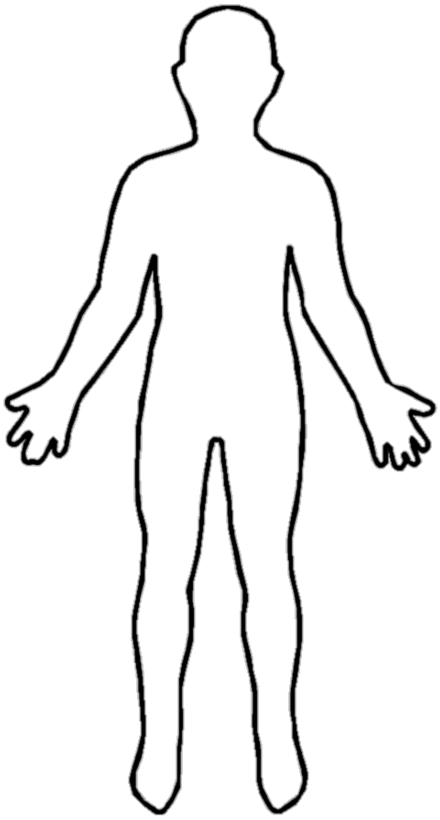

☐ **No discomfort.**

DAY 5

Exercise 5E

Positive Experience 1 (short title):

Positive Meaning/ Outcomes

Positive Experience 2 (short title):

Positive Meaning/ Outcomes

Positive Experience 3 (short title):

Positive Meaning/ Outcomes

Exercise 7B

Day 5 Altruism Task

Who did you help:

How did you help:

How much time did you spend on this task:

☐ Less than 1 hour

☐ Between 1-2 hours

☐ Between 2-5 hours

☐ Half of the day

☐ All day

DAY 6

Exercise 1C

Who did you bring today:

Exercise 2E

One Purpose:

Could it:

1. …bring others joy?	YES	NO
2. … make someone smile?	YES	NO
3. … make people laugh?	YES	NO
4. … make others change for good?	YES	NO
5. … make people feel strong?	YES	NO
6. … contribute to a good cause?	YES	NO
7. … provide food to others?	YES	NO
8. … spread knowledge?	YES	NO
9. … bring peace?	YES	NO
10. … bring development?	YES	NO

One Step:

Exercise 3C

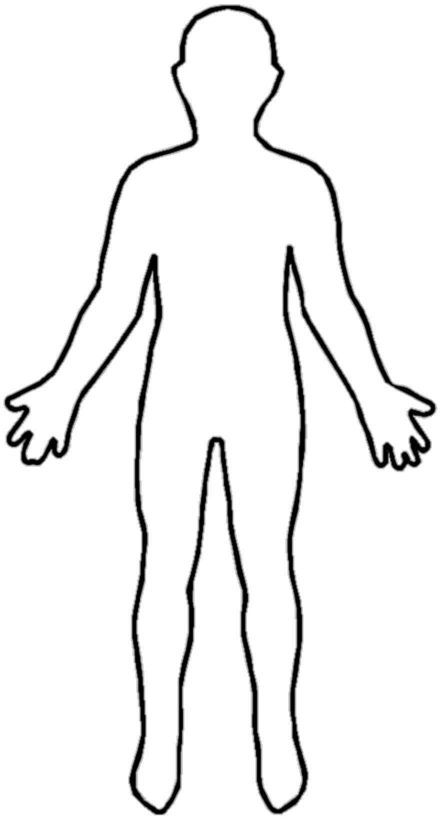

☐ **No discomfort.**

Exercise 5F

External Resources	Internal Resources
1.	1.
2.	2.
3.	3.

Exercise 7B

Day 6 Altruism Task

Who did you help:

How did you help:

How much time did you spend on this task:

☐ Less than 1 hour

☐ Between 1-2 hours

☐ Between 2-5 hours

☐ Half of the day

☐ All day

DAY 7

Exercise 1C

Who did you bring today:

Exercise 2I

One Gesture:

Exercise 3C

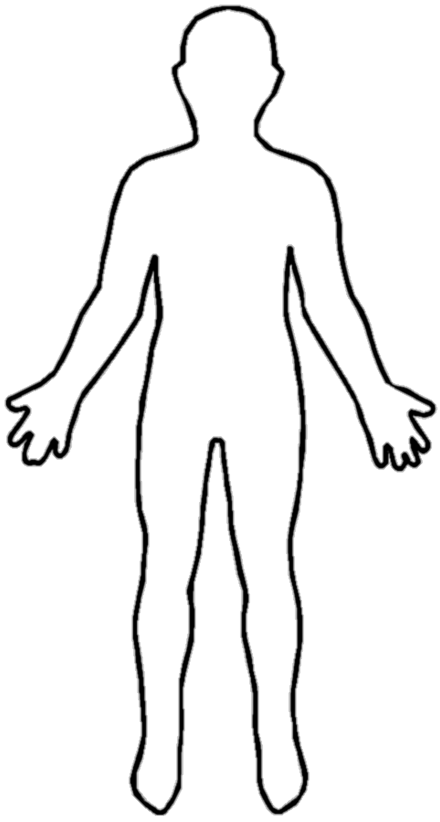

☐ **No discomfort.**

Exercise 5G

**Big**	**Everyday**
**1.**	**1.**
**2.**	**2.**
**3.**	**3.**
**4.**	**4.**
**5.**	**5.**

Exercise 7B

Day 7 Altruism Task

Who did you help:

How did you help:

How much time did you spend on this task:

- ☐ Less than 1 hour
- ☐ Between 1-2 hours
- ☐ Between 2-5 hours
- ☐ Half of the day
- ☐ All day

Thank you for your participation!
