# Supplementary material for: Advancing Posttraumatic Stress Disorder Diagnosis and the Treatment of Trauma in Humanitarian Emergencies via Mobile Health: Protocol for a Proof-of-Concept Nonrandomized Controlled Trial
Source: JMIR Res Protoc. 2022 Jun 15;11(6):e38223. doi: 10.2196/38223 (PMC9244657; doi:10.2196/38223)
Supplement: Multimedia Appendix 6 [file resprot_v11i6e38223_app6.pdf]

THE UNIVERSITY OF SYDNEY

Brain and Mind Centre
Sydney Medical School

100 Mallett Street
CAMPERDOWN NSW 2050
NSW 2006 AUSTRALIA

Web: http://www.sydney.edu.au/

Cognitive and Electrophysiological Responses
to a Trauma Intervention for Humanitarian Emergencies.

PARTICIPANT INFORMATION STATEMENT
QUEENSLAND

1. Purpose of the study

This is an invitation to participate in a study by researchers at the University of Sydney. The purpose of the research is to investigate how African adults (20-50 years old) traumatized by war respond to a new intervention (a set of take-home audio exercises on a mobile smartphone App) aimed to reduce symptoms of trauma, and how brain mechanisms and cognitive processes (for example: memory, attention, and problem solving) may be impacted by war trauma. Individuals that have been traumatized often struggle with nightmares (bad dreams), flashbacks, intrusive thoughts, discomfort in their bodies, avoidance, profound sadness, among other symptoms. Today, it is possible to detect some of these symptoms by studying the brain.

With the help of Africans that have been exposed to war, we seek to better understand if a treatment based on a neurophysiological framework as proposed in this study is effective for African populations that survive humanitarian emergencies. By recording your brain activity before and after using the experimental App mobile audio intervention, we will be able to detect changes that could indicate if the intervention we are testing works. The information we collect from you will also help us study how war trauma may impact thinking, memory, planning, and other functions in the brain. If the researchers can detect enough evidence that the intervention works, this project could contribute to bring this new trauma relief method free of charge to those in conflict and emergency zones without access to doctors and treatment centers in Africa and around the world.

To conduct this investigation, we are recruiting 60 Africans: 30 currently experiencing trauma that meet the criteria for Post-Traumatic Stress Disorder (PTSD), and 30 with no trauma symptoms. You have been identified as a possible participant in this study because you meet our criteria as an African that fled during a humanitarian emergency in your home country and currently resides in Australia.

Who is Doing the Study?

The study is being led by Assoc. Prof Jim Lagopoulos (Associate professor of Cognitive Neuroimaging). Other members of the research team include Dr. Brian O'Toole, Clinical Senior Lecturer in Psychiatric Epidemiology, Miss Janaina Pinto, PhD candidate at the Brain and Mind Centre, Miss Grace Forsyth, Judith Ocokuru, and Cyrana Gallay, research assistants, Dr Marta Garrido, Computational Cognitive Neuroscience Laboratory Group Leader at the Queensland Brain Institute, Dr Ilvana Dzafic, Queensland Brain Institute Postdoctoral researcher, and Dr Clare Harris, Queensland Brain Institute Masters candidate.

2. What does the study involve?

If you choose to take part in this study you will be asked to be visited at home for consenting and interviews, attend two (2) visits to the Sunshine Coast Mind and Neuroscience – Thompson Institute, as well as completing 7 consecutive days of 90-100-minute audio exercises at home via a mobile smartphone App in addition to 15 minutes of self-report questionnaires between both visits, and a 30-40 minute phone interview after 30 days. You will receive a smartphone with the App containing audio exercises on your first visit, and return it to us on your second visit. We will cover your transportation costs to the centre.

2. Details for your Home Visit (Apr. 2 hours):
2.a) Consent (5 minutes):
The researcher will read and review this information sheet in person with you, answer any questions or concerns you may have, and make sure you understand the study before you decide if you would like to participate. If you agree to participate, you will fill out and sign the consent form attached, and commence the tasks outlined below.

2.b) Diagnostic Assessments and Interviews (1:15-1:45 hours):
You will complete a psychiatric assessment and several interviews. You will be asked questions about your ethnic background, family history, migration to Australia, traumatic events, and other psychological symptoms you may or may not have experienced in your life. During this stage, the researcher will confirm if you meet the diagnostic criteria for PTSD. Since both Africans with and without PTSD are being recruited to complete the same tasks in this study, a diagnosis (or lack of) will not change your eligibility to participate. You may choose to not answer a question if it makes you feel uncomfortable. All information you give will be strictly confidential. There will be only one researcher interviewing you. A research assistant may observe the interviews for training purposes if you feel comfortable. We will always ask for your approval before inviting someone to join.

3. Details for your First Visit to the Sunshine Coast Mind and Neuroscience – Thompson Institute for Baseline Assessments (Apr. 2:30 hours):
3.a) Cognitive Activities (1 hour):
You will complete several tasks on an iPad touch screen. The tasks are similar to playing video games. The iPad will be recording how your brain performs on each task based on your responses. You will be given detailed instructions on how to complete each task.

3.b) Brain Activity Recording (1hour):
We will collect data on your brain activity via electroencephalography, a method that shows the waves of your brain (sometimes called EEG). It is a painless procedure that involves placing a cap on your head as well as some recording disks on your face. The researcher will then put some gel into the recording disks of the cap. Once the cap is in place, the researcher will ask you to sit still and close your eyes for 3 minutes while your brain activity is being recorded. After that, you will be asked to remain seating still for another 3 minutes with your eyes open. When all recording is done, you will be given a towel so you can wash the gel off your hair.

3.c) App Instructions (10 minutes):
Prior to leaving the Thompson Institute, you will receive a kit that you will take home for 7 days. The researcher will go over all contents in the kit and give you detailed instructions on how to use each: (1) the android smartphone with the App installed and charger; (2) a stress thermometer; (3) two pairs of headphones; (4) 10 biodot finger stickers; and (5) printed instructions and (6) a response booklet for the exercises. Preliminary information needed to complete the tasks at home will also be collected during this time.

4. Details for the intervention we will ask you to do at home on your own (90-100 minutes per day over 7 consecutive days), and an additional 15 minutes during the week for questionnaires:

4.a) App Intervention (90-100 minutes per day):
You will be choosing a time of your day at home when you can reserve 90-100 minutes each day to practice the audio exercises on the smartphone App. During the exercises, you will tape a stress thermometer and a biodot sticker (both provided) to two of your fingers to track your temperature during the 90-100 minutes. Both tools are painless, harmless, and easy to set-up.

The exercises are split into seven daily blocks, and are also easy to complete:

- Block 1: Three (3) daily exercises that involve breath work and imagination.
- Block 2: One (1) daily exercise that involve thinking about relationships and connection with friends, family, or strangers.
- Block 3: Four (4) daily exercises that involve guided visualisation, tapping with sounds of drums before and after thinking of a scene and the good thought that comes with it.
- Block 4: Two (2) daily exercises that involve imagination, mindful breathing, and making vocal sounds.
- Block 5: Two (2) daily exercises that involve thoughts you have about yourself and the world; how you think and feel about yourself and bad things that may have happened to you; what brings you fear and anxiety, and the ability to reflect on your present moment, instead of the past or future.
- Block 6: One (1) daily exercises that involve breathing and relaxing.
- Block 7: In the final block, you are assignment a daily altruism task to be completed between sessions, and during sessions 2-7, you will be writing about the task you performed the previous day.

You will receive a response booklet to take home to write answers on during the exercises. You will return the booklet to the researchers during your second visit to the Thompson Institute in the Sunshine Coast. While the booklet will be securely stored at our research centre, all personal information you write in this booklet will be kept confidential and will not be published in the study results.

4.b) Self-Report Questionnaires (15 minutes total):
Before returning to the Thompson Institute in the Sunshine Coast for your second visit, you will be asked to complete three short questionnaires at home to assess personality and resiliency traits, alongside any difficulties you may have had after migrating to Australia.

5. Details for your Second Visit to the Sunshine Coast Mind and Neuroscience – Thompson Institute for Post Treatment Assessments (2:30 hours):
After the 7 days, you will return to the Thompson Institute in the Sunshine Coast for your second first. You will return the smartphone and kit, and we will repeat the cognitive activities (3.c) and the brain activity recording (3.d), as described above. You will also repeat short interview about trauma symptoms you may have experienced during the past week. Like the first visit, we will cover your transportation costs to the centre.

5. Details for Telephone Interview (30-40 minutes):
Thirty (30) days after your last visit to the Sunshine Coast Mind and Neuroscience – Thompson Institute, we will schedule a phone call with you to ask you questions about PTSD symptoms you may have experienced in the past month.

6. Can I withdraw from the study?
You can choose to withdraw from the study at any time. Your decision whether to participate will not affect your future relations with The University of Sydney, the Brain and Mind Centre, Sunshine Coast Mind and Neuroscience – Thompson Institute, The University of Queensland or Queensland Brain Institute. We will share with you if any information becomes available during the study that might be relevant to your willingness to continue participation. If you withdraw during stage 2, arrangements will be made to return the smartphone and kit that you will be given on the first visit, and any costs that might be involved to return the equipment will be covered.

7. Will anyone else know the results?
Only the researchers conducting this study, and regulatory authorities of human ethics research that verifies clinical trial procedures will be granted direct access to your records, however your confidentiality will not be violated. If the results of this study are published, your name will not be published in it, so that your identity remains confidential.

8. Can I tell other people about the study?
Yes, you can tell your friends about the trial. If they are interested they can contact our research clinic at

+61 481 244 463 or jpin6516@uni.sydney.edu.au.

9. What if I require further information about the study or my involvement in it?
When you have read this information, the researcher will discuss it with you further and answer any questions you may have. If you would like to know more at any stage, please feel free to contact Miss Pinto at +61 481 244 463 or jpin6516@uni.sydney.edu.au.

10. What if I have a complaint or any concerns?
This study has been reviewed by the University of Sydney's Human Research Ethics Committee. Any person with concerns or complaints about the conduct of a research study can contact The Manager, Human Ethics Administration, University of Sydney on +61 2 8627 8176 (Telephone); +61 2 8627 8177 (Facsimile) or ro.humanethics@sydney.edu.au (Email).

11. Are there any risks involved in the study?
There are no major health risks in participating in this study. You may feel tired during the initial interviews (2.b) due to its length, and some of the questions may make you anxious or upset by remembering and talking about events that happened in your life. If you feel uncomfortable, you can choose not to answer a question. If you have a history of trauma and meet the diagnostic criteria for PTSD, you may feel anxious and confused about what it means. A researcher will be available to explain how that might impact your life, and refer you to treatment and resources to help you learn about the condition. If you have PTSD there is also a possibility that by answering questions about what happened to you during the war, you might feel anxious and experience traumatic symptoms. If you do, the interview will be interrupted and a researcher certified in trauma relief will be available to help you alleviate the symptoms on site. If the symptoms persist, we will refer you to a free treatment program outside of the Thompson Institute.

During cognitive activities (3.c), you may feel tired or bored if you do not enjoy the tasks/ games on the iPad. The EEG brain activity recording (3.d) is a risk-free method of collecting brain data, but you may feel discomfort while sitting in a chair for 60 minutes, and upon completion you may not enjoy to wash the gel off your hair. The gel does not cause harm to your hair or scalp and comes off with water. The intervention App was designed as a safe tool to promote a sense of well-being in individuals with or without trauma and there are no known major health risks involved in practicing the exercises. However, if you have a history of trauma and a current diagnosis of PTSD, it is possible that you become bothered by trauma symptoms during the exercises. Should you feel distressed by unpleasant symptoms of trauma while you are using the App (for example, having bad dreams, flashbacks or body discomfort) or need to speak with someone, inform the researcher via the App direct call icon and contact the following agencies to receive free psychological assistance:

- Queensland Program of Assistance for Survivors of Torture and Trauma (QPASTT):
Contact phone: (07) 3391 6677; E-mail: admin@qpastt.org.au
Address: 28 Dibley Street, Woolloongabba, QLD 4102

- Mater Refugee Complex Care Clinic (MRCC):
Contact phone: (07) 3163 8111; E-mail: mrccc@mater.org.au
Address: Level 4, Salmon Building, Raymond Terrace, South Brisbane 4101

- Refugee Health Connect (RHC):
Contact phone: (07) 3864 7580; E-mail: refugeehealth@bsphn.org.au

If you present symptoms of PTSD, you may be referred for further assessment and treatment should you provide consent. You can request referral for trauma counselling at any time and finish the program, but in this scenario your data cannot be used further in the analysis.

12. Are there any benefits involved in the study?
You will receive free comprehensive psychiatric and cognitive performance assessments. There are no out of pocket costs associated with participating in this study.

13. Do I have to do the study?
No, you are free to decide on your own if you would like to participate.

14. Removal from the Study
For your safety, if you exhibit signs of suicide or homicide risk, drug or alcohol abuse, or if you present other serious health needs that require immediate professional psychological assistance, you will be removed from the study and referred to free and confidential professional care at Queensland Program of Assistance for Survivors of Torture and Trauma (QPASTT), or the Mater Refugee Complex Care Clinic (MRCC).

15. Can I know the results?
Yes. If you would like to know the results of your tests, the researcher can discuss them with you in person during your second visit to the Sunshine Coast Mind and Neuroscience – Thompson Institute. Please note that all diagnostic and cognitive testing information we can provide is for research purposes, and we recommend further clinical tests by licensed professionals to confirm all results. We ask that you plan to stay at the Thompson Institute for an additional twenty minutes during your second visit if you would like to review your results.

16. Do I get anything for being in this study?
If you choose to participate and complete the study, you will receive a Coles Supermarket gift card as a compensation for your time. Food and transportation costs for both visits to the Sunshine Coast Mind and Neuroscience – Thompson Institute will also be covered. You will not be able to keep the mobile smartphone with the App or any other items included in the kit after you conclude your participation.

This information sheet is for you to keep. Thank you.
