# Supplementary material for: Advancing Posttraumatic Stress Disorder Diagnosis and the Treatment of Trauma in Humanitarian Emergencies via Mobile Health: Protocol for a Proof-of-Concept Nonrandomized Controlled Trial
Source: JMIR Res Protoc. 2022 Jun 15;11(6):e38223. doi: 10.2196/38223 (PMC9244657; doi:10.2196/38223)
Supplement: Multimedia Appendix 8 [file resprot_v11i6e38223_app8.pdf]

Prof Caroline Hunt
School of Psychology
M02F - Mallett Street Campus
The University of Sydney
NSW 2006 Australia

PARTICIPANT INFORMATION STATEMENT QUEENSLAND

Invitation for Follow-Up Interviews

Cognitive and Electrophysiological Responses to a Trauma Intervention for Humanitarian Emergencies

1. Purpose of the Follow-Up

This is an invitation to all who participated in our clinical trial *"Cognitive and Electrophysiological Responses to a Trauma Intervention for Humanitarian Emergencies*" in 2019 to enroll on a follow-up series of interviews post the 7-day App intervention at three (3) time periods. The purpose of the follow-up is to investigate the duration of symptom improvement reported during the first phase of the project. Understanding the duration of efficacy of the App intervention will assist researchers in further understanding the mechanisms of trauma healing, and identifying the populations in humanitarian emergencies around the world who can best benefit from the program.

Who is Doing the Follow-up?

The follow-up is being led by Professor Caroline Hunt, Head of the Clinical Psychology Unit at the University of Sydney. Other members of the research team include Miss Janaina Pinto, PhD candidate at the Brain and Mind Centre, and Dr. Brian O'Toole, Clinical Senior Lecturer in Psychiatric Epidemiology.

What does the follow-up involve?

If you choose to take part in the follow-up phase of the study, you can choose between: (1) Completing interviews via phone calls, or (2) requesting to meet in person at your home or another location where you feel comfortable meeting with our researcher. The follow-up phase does not include repeating the App intervention week, another visit to the Sunshine Coast to collect electrophysiological data (brain waves recording) or cognitive tests (tablet games). Only interviews will be conducted.

2. Details for the Follow-Up Interviews

2.a) Consent (10 minutes):

After receiving and reading a copy of this statement, the researcher will read and review this information sheet with you in person, via telephone or video call, answer any questions or concerns you may have, and make sure you understand the study before you decide if you would like to participate. If you agree to participate, you will fill out and sign the consent form attached, and schedule with the researcher the interviews below. The signed consent form can be returned by mail, e-mail with a digital signature, or be arranged to be picked-up in person.

2.b) Clinical Assessments and Interviews (30-60 minutes at 3 time periods, total*: 1:30-3hours*):
You will complete a series of clinical interviews at 3 different stages: at 3, 6, and 12 months after the date of your last visit to the Thompson Institute in the Sunshine Coast. Dates vary by participant; your exact dates can be provided by the researcher before you decide to sign the consent. The researcher will repeat a few of the same interviews (on trauma, depression, physical symptoms, as well as resilience and mindfulness abilities) that you already completed and are familiar with during your first and second visits to the institute. You may choose to not answer a question if it makes you feel uncomfortable. All information you give will be strictly confidential. There will be only one researcher interviewing you. If you choose to complete the interviews in person, a research assistant may observe the interviews for training purposes if you feel comfortable. We will always ask for your approval before inviting someone to join.

3. **Can I withdraw from the study?**
You can choose to withdraw from the follow-up phase at any time. Your decision whether to participate will not affect your future relations with The University of Sydney. We will share with you if any information becomes available during the study that might be relevant to your willingness to continue participation.

4. **Will anyone else know the results?**
Only the researchers conducting this study, and regulatory authorities of human ethics research that verifies clinical trial procedures will be granted direct access to your records, however your confidentiality will not be violated. If the results of this study are published, your name will not be published in it, so that your identity remains confidential.

5. **Can I tell other people about the study?**
You are welcome to share with others about your involvement in the project, but unfortunately, we cannot enroll new participants. This follow-up phase of the study is only for participants that already completed the App clinical trial in 2019.

6. **What if I require further information about the study or my involvement in it?**
When you have read this information, the researcher will discuss it with you further and answer any questions you may have. If you would like to know more at any stage, please feel free to contact Miss Pinto at +61 481 244 463 or jpin6516@uni.sydney.edu.au.

7. **What if I have a complaint or any concerns?**
This study has been reviewed by the University of Sydney's Human Research Ethics Committee. Any person with concerns or complaints about the conduct of a research study can contact The Manager, Human Ethics Administration, University of Sydney on +61 2 8627 8176 (Telephone); +61 2 8627 8177 (Facsimile) or ro.humanethics@sydney.edu.au (Email).

8. **Are there any risks involved in the study?**
There are no major health risks in participating in this study. You may feel anxious or upset by remembering and talking about events that happened in your life. If you feel uncomfortable, you can choose not to answer a question. If you have a history of trauma and meet the diagnostic

criteria for PTSD, you may feel anxious and confused about what it means. A researcher will be available to explain how that might impact your life, and refer you to treatment and resources to help you learn about the condition. If you have PTSD there is also a possibility that by answering questions about what happened to you during the war, you might feel anxious and experience traumatic symptoms. If you do, the interview will be interrupted and a researcher certified in trauma relief will be available to help you alleviate the symptoms in person or via telephone or video call. If the symptoms persist, we will refer you to a free treatment program:

- **Queensland Program of Assistance for Survivors of Torture and Trauma (QPASTT):**
  Contact phone: (07) 3391 6677; E-mail: admin@qpastt.org.au
  Address: 28 Dibley Street Woolloongabba, QLD 4102

- **Mater Refugee Complex Care Clinic (MRCC):**
  Contact phone: (07) 3163 8111; E-mail: mrccc@mater.org.au
  Address: Level 4, Salmon Building, Raymond Terrace, South Brisbane 4101

- **Refugee Health Connect (RHC):**
  Contact phone: (07) 3864 7580; E-mail: refugeehealth@bsphn.org.au

9. Are there any benefits involved in the study?
You will receive a free follow-up of your psychiatric assessments at 3, 6, and 12-months. There are no out of pocket costs associated with participating in this study.

10. Do I have to do the follow-up interviews?
No, you are free to decide on your own if you would like to participate in this new phase of our clinical trial.

11. Removal from the Study
For your safety, if you exhibit signs of suicide or homicide risk, drug or alcohol abuse, or if you present other serious health needs that require immediate professional psychological assistance, you will be removed from the follow-up and referred to free and confidential professional care at Queensland Program of Assistance for Survivors of Torture and Trauma (QPASTT), or the Mater Refugee Complex Care Clinic (MRCC).

12. Can I know the results?
Yes. If you would like to know the results of your interviews, the researcher can discuss them with you in person or on the phone. Please note that all diagnostic testing information we can provide is for research purposes, and we recommend further clinical tests by licensed professionals to confirm all results.

13. Do I get anything for being in this study?
If you choose to participate and complete the study, you will receive a Coles Supermarket gift card per visit or per phone call as a compensation for your time.
