# Supplementary material for: Advancing Posttraumatic Stress Disorder Diagnosis and the Treatment of Trauma in Humanitarian Emergencies via Mobile Health: Protocol for a Proof-of-Concept Nonrandomized Controlled Trial
Source: JMIR Res Protoc. 2022 Jun 15;11(6):e38223. doi: 10.2196/38223 (PMC9244657; doi:10.2196/38223)
Supplement: Multimedia Appendix 9 [file resprot_v11i6e38223_app9.pdf]

Prof Caroline Hunt
School of Psychology
M02F - Mallett Street Campus
The University of Sydney
NSW 2006 Australia

PARTICIPANT CONSENT FORM

I, ... [PRINT NAME], agree to participate in the follow-up phases of the research project titled: Cognitive and Electrophysiological Responses to a Trauma Intervention for Humanitarian Emergencies.

In giving my consent I acknowledge that:

1. The procedures required for the project and the time involved for participation in the project has been explained to me, and any questions I have about the project have been answered to my satisfaction.

2. I have read the Information Statement and have been given the opportunity to discuss the information and my involvement in the project with the researcher/s.

3. I understand that being in the follow-up phase of the study I already completed is completely voluntary – I am not under any obligation to consent to my participation.

4. I understand that my involvement is strictly confidential. I understand that research data gathered from the results of the study may be published however no information about myself will be used in any way that is identifiable.

5. I understand that I can withdraw from the study at any time without prejudice to my relationship with the researcher/s or the University of Sydney or the wider community in the future.

6. I understand that all assessments can be stopped at any time if I do not wish to continue. In such case, all information provided will not be included in the study.

7. I understand that if I present the need for professional psychological assistance, the researchers will refer me to free health services at NSW Service for the Treatment and Rehabilitation of Torture and Trauma Survivors (STARTTS), the NSW Refugee Health Service (RHS), or to the Asylum Seekers Centre (ASC).

8. I consent to share my clinical results, including mental health symptoms with staff at the NSW Service for the Treatment and Rehabilitation of Torture and Trauma Survivors (STARTTS), NSW Refugee Health Service (RHS), Asylum Seekers Centre (ASC), or a local hospital in case of treatment referral or emergency.

9. I consent to Receiving Feedback of the follow-up interviews: YES ☐ NO ☐
   Send it to:

   Address: .... E-mail: ....

   ....

....

Please PRINT name Signature of Participant Date
